# Molecular features and clinical implications of the heterogeneity in Chinese patients with HER2-low breast cancer

**DOI:** 10.1038/s41467-023-40715-x

**Published:** 2023-08-22

**Authors:** Lei-Jie Dai, Ding Ma, Yu-Zheng Xu, Ming Li, Yu-Wei Li, Yi Xiao, Xi Jin, Song-Yang Wu, Ya-Xin Zhao, Han Wang, Wen-Tao Yang, Yi-Zhou Jiang, Zhi-Ming Shao

**Affiliations:** 1 Department of Breast Surgery, Fudan University Shanghai Cancer Center, Shanghai, 200032 China; 2 Key Laboratory of Breast Cancer in Shanghai, Fudan University Shanghai Cancer Center, Shanghai, 200032 China; 3 Department of Oncology, Shanghai Medical College, Fudan University, Shanghai, 200032 China; 4 Department of Pathology, Fudan University Shanghai Cancer Center, Shanghai, 200032 China

**Keywords:** Breast cancer, Targeted therapies, Cancer genomics, Tumour heterogeneity

## Abstract

The molecular heterogeneity and distinct features of HER2-low breast cancers, particularly in the Chinese population, are not well understood, limiting its precise management in the era of antibody‒drug conjugates. To address this issue, we established a cohort of 434 Chinese patients with HER2-low breast cancer (433 female and one male) and integrated genomic, transcriptomic, proteomic, and metabolomic profiling data. In this cohort, HER2-low tumors are more distinguished from HER2-0 tumors in the hormone receptor–negative subgroup. Within HER2-low tumors, significant interpatient heterogeneity also exists in the hormone receptor–negative subgroup: basal-like tumors resemble HER2-0 disease, and non-basal-like HER2-low tumors mimic HER2-positive disease. These non-basal-like HER2-low tumors are enriched in the HER2-enriched subtype and the luminal androgen receptor subtype and feature *PIK3CA* mutation, *FGFR4/PTK6/ERBB4* overexpression and lipid metabolism activation. Among hormone receptor–positive tumors, HER2-low tumors show less loss/deletion in 17q peaks than HER2-0 tumors. In this work, we reveal the heterogeneity of HER2-low breast cancers and emphasize the need for more precise stratification regarding hormone receptor status and molecular subtype.

## Abstract (web summary)

HER2-low breast cancer has recently been defined as a potential subtype for sensitivity to novel antibody-drug conjugates. Here, the authors analyse a multiomics cohort of 434 HER2-low patients and find an altered molecular status compared to other subtypes and the interpatient heterogeneity within this subtype.

## Introduction

Breast cancer is the most common malignancy in women worldwide and in China^1,2^. Breast cancers can be subdivided into several subtypes according to hormone receptors (HRs, including the estrogen receptor and the progesterone receptor) and human epidermal growth factor receptor 2 (HER2, or *ERBB2*) status. HER2 is a major driving molecule and a therapeutic target of breast cancer, and HER2 status is determined by immunohistochemistry (IHC) and in situ hybridization (ISH) methods in the clinic^3,4^. Although HER2-positive diseases (defined as IHC 3+ or 2+ with ISH+) can benefit from anti-HER2 targeted therapies, they account for only 15–20% of all breast cancers^5^. On the other hand, many more patients bearing tumors with low to moderate levels of HER2, which are called HER2-low breast cancers (IHC 1+ or 2+ with ISH–), have not been considered candidates for conventional anti-HER2 therapies during the past two decades.

Intriguingly, recent advances regarding anti-HER2 antibody‒drug conjugates (ADCs) have shown promising effects in HER2-low breast cancers^6–8^. The results from the DESTINY-Breast04 trial proved the efficacy and safety of trastuzumab deruxtecan (T-DXd) in HER2-low breast cancers, where heavily pretreated advanced HER2-low breast cancer patients achieved significantly improved survival compared with chemotherapy-treated patients^8^. These results expand our knowledge on the subtyping and treatment of breast cancer, which also emphasizes the importance of this subgroup of patients in future practice.

With the evolution of clinical practice, a thorough and updated molecular description of this subgroup of patients (beyond the binary HER2 status classification) is needed; these findings may suggest a discrepancy in drug response and support more precise management in the future. However, although several previous studies have tried to characterize HER2-low breast cancers, studies based on both reliable HER2-low diagnosis and comprehensive multiomics profiling are lacking. Critical topics such as whether HER2-low breast cancer is a distinct entity and what landscape of heterogeneity is present within HER2-low breast cancers remain obscure^6,7,9^. Moreover, although marked ethnic and race disparities in breast cancer have been observed^10^ and have also been inferred from the subgroup analysis in the DESTINY-Breast04 trial^8^, whether patients with HER2-low breast cancers may exhibit these differences is not yet clear. These unknowns are factors limiting more precise patient selection and application of better drug combinations for HER2-low patients in the new era.

In this study, we established an original multiomics cohort containing 434 HER2-low breast cancers to characterize HER2-low breast cancers in the Chinese population both clinically and molecularly. We compared HER2-low breast cancers with HER2-0 and HER2-positive diseases to investigate whether HER2-low tumors are indeed a distinct entity. We further revealed the interpatient heterogeneity, potential driving mechanisms and therapeutic targets within HER2-low breast cancers. We also focused on the ethnic and race difference of HER2-low breast cancers and discussed the potential effect it might have in clinical practice. Our data and findings add to the understanding of HER2-low breast cancers and may benefit future practice.

## Results

### Study design and cohort information

**Fig. 1:**
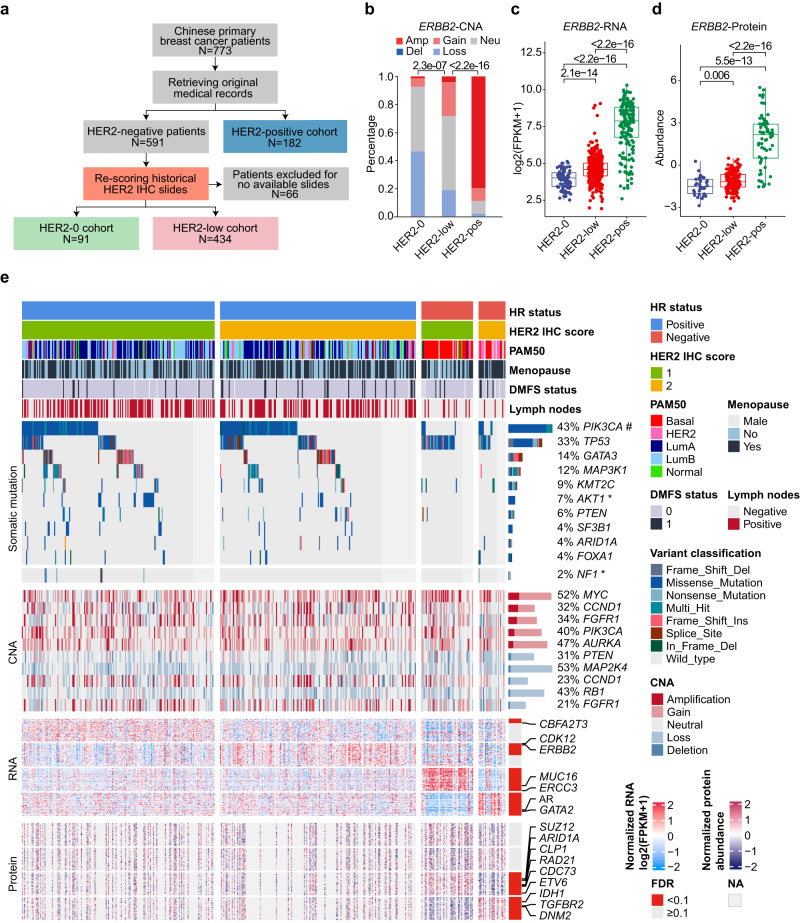
The design and molecular landscape of the Fudan University Shanghai Cancer Center (FUSCC) HER2-low breast cancer cohort. **a** Flowchart of the patient selection process and stratification. **b** Bar plot comparing the copy number alterations (CNAs) of *ERBB2* among HER2 status subgroups based on Genomic Identification of Significant Targets in Cancer (GISTIC) analysis. Amp: 2, gain: 1, neu: 0, loss: −1, del: −2. *P* values were computed using the two-sided Fisher’s exact test. **c**, **d** Boxplots comparing the RNA (**c**) and protein (**d**) levels of *ERBB2* among HER2-0 (*N* = 88 for RNA and 34 for protein), HER2-low (*N* = 421 for RNA and 156 for protein) and HER2-positive (*N* = 181 for RNA and 64 for protein) tumors. The centerline represents the median, the box limits represent the upper and lower quartiles, the whiskers represent the 1.5× interquartile range, and the points represent individual samples. *P* values were computed using the two-sided Wilcoxon test. **e** Molecular landscape of HER2-low breast cancers stratified by hormone receptor (HR) status and HER2 IHC score. Genes marked with * and # represent genes differentially mutated between HER2 1+ and HER2 2+ patients in the HR-positive and HR-negative subgroups, respectively. Detailed criteria for gene screening and annotation are provided in the Methods. Source data are provided as a Source Data file.

We reviewed all early-stage primary breast cancer patients who underwent breast surgery at Fudan University Shanghai Cancer Center (FUSCC) between January 1, 2013, and December 31, 2014. A total of 773 eligible patients were initially included. Then, we collected their HER2 IHC score and ISH status data from archived pathology reports. To classify HER2-low breast cancer accurately, for all patients with historical HER2-negative diagnosis (i.e., HER2 0, HER2 1+, and HER2 2+ with ISH–) (*N* = 591), we rescored their historical HER2 IHC slides via rigorous methods according to the latest 2018 ASCO/CAP guideline^11^ (Fig. 1a). In the rescoring, 66 patients were excluded because of no available historical HER2 IHC slides. Finally, we confirmed 434 HER2-low breast cancers and 91 HER2-0 breast cancers in our cohort, together with 182 HER2-positive breast cancers (Methods).

Clinical information, including clinicopathological features, treatment information and follow-up records of all included patients, was extracted from medical records. Collected tumor samples underwent whole-exome sequencing (WES), OncoScan CNA assay, RNA sequencing, tandem mass tag (TMT) quantitative proteomics and metabolomics profiling to generate multiomics data (Supplementary Fig. S1a, Methods and Supplementary Methods).

In our multiomics dataset, HER2-low tumors contained only a low rate of *ERBB2* amplification (4.0%) (Fig. 1b). The RNA and protein levels of *ERBB2* in HER2-low tumors were significantly higher than those in HER2-0 tumors and lower than those in HER2-positive tumors (all *P* < 0.05, Wilcoxon test, Fig. 1c, d). Similar results were also observed after stratification by HR status in our cohort (Supplementary Fig. S1b–d) and other datasets^9,12,13^. Figure 1e displays the molecular landscape of HER2-low breast cancers stratified by HR status and HER2 IHC scores. For genomic alterations, recurrent mutations in breast cancer, such as *PIK3CA*, *TP53*, and *GATA3*, were found in HER2-low breast cancers, and the downstream effects of copy number alterations (CNAs) can also be seen at the RNA and protein levels (Supplementary Fig. S2). In addition, at the transcriptome, proteome, and metabolome levels, the pattern in the heatmaps showed interpatient heterogeneity within HER2-low breast cancers, and there were more differences between HER2 1+ tumors and HER2 2+ tumors in the HR-negative subgroup than in the HR-positive subgroup (Fig. 1e and Supplementary Fig. S3).

### Clinicopathological characteristics of the FUSCC HER2-low breast cancer cohort

For baseline clinicopathological features, HR positivity was more common in HER2-low patients than in HER2-0 and HER2-positive patients (83.2%, 69.2% and 55.5%, respectively, *P* = 7.8e–12, *χ*^2^ test). HER2-low patients also had fewer grade 3 tumors than HER2-0 and HER2-positive patients (38.3%, 51.1% and 68.0%, respectively, *P* = 1.9e–10, *χ*^2^ test) (Supplementary Table S1). Considering the discrepancy between HR-positive and HR-negative breast cancers in terms of both molecular nature and clinical management, we stratified patients by HR status to characterize HER2-low breast cancers among luminal and triple-negative breast cancers (TNBCs) separately, and no significant differences were found regardless of HR status in terms of clinicopathological characteristics (Table 1).

**Table 1:** Baseline clinicopathological characteristics of HER2-low and HER2-0 breast cancers stratified by hormone receptor (HR) status

	HR-positive			HR-negative		
	HER2-0	HER2-low	*P*	HER2-0	HER2-low	*P*
	*N* = 63	*N* = 361		*N* = 28	*N* = 73	
**Age (years)**						
Mean (SD)	52.51 (12.12)	53.17 (10.41)	0.650	55.96 (11.41)	53.74 (10.93)	0.368
**Age (years, %)**						
<40	10 (15.9)	30 (8.3)	0.171	2 (7.1)	3 (4.1)	0.679
40–59	36 (57.1)	233 (64.5)		17 (60.7)	50 (68.5)	
≥60	17 (27.0)	98 (27.1)		9 (32.1)	20 (27.4)	
**Menopause (%)**						
No	27 (42.9)	156 (43.2)	1	6 (21.4)	28 (38.9)	0.108
Yes	36 (57.1)	204 (56.5)		22 (78.6)	44 (61.1)	
Male	0 (0.0)	1 (0.3)		0 (0.0)	0 (0.0)	
NA	0	0		0	1	
**Laterality (%)**						
Left	34 (54.0)	182 (50.4)	0.682	14 (50.0)	35 (47.9)	1
Right	29 (46.0)	179 (49.6)		14 (50.0)	38 (52.1)	
**HER2 IHC score (%)**						
HER2 0	63 (100.0)	0 (0.0)	–	28 (100.0)	0 (0.0)	–
HER2 1+	0 (0.0)	179 (49.6)		0 (0.0)	48 (65.8)	
HER2 2+	0 (0.0)	182 (50.4)		0 (0.0)	25 (34.2)	
**Histology (%)**						
IDC	62 (98.4)	343 (95.0)	0.235	26 (92.9)	65 (89.0)	1
ILC	0 (0.0)	14 (3.9)		0 (0.0)	2 (2.7)	
Other	1 (1.6)	4 (1.1)		2 (7.1)	6 (8.2)	
**Grade (%)**						
<3	41 (67.2)	245 (71.4)	0.542	2 (7.4)	5 (8.1)	1
3	20 (32.8)	98 (28.6)		25 (92.6)	57 (91.9)	
NA	2	18		1	11	
**Ki67 percentage (%)**						
<20	20 (31.7)	110 (30.5)	0.883	0 (0.0)	2 (2.7)	1
≥20	43 (68.3)	251 (69.5)		28 (100.0)	71 (97.3)	
**sTILs**						
Mean (SD)	0.16 (0.12)	0.13 (0.11)	0.106	0.24 (0.21)	0.25 (0.19)	0.776
**iTILs**						
Mean (SD)	0.02 (0.02)	0.01 (0.01)	0.636	0.04 (0.04)	0.04 (0.04)	0.614
**pT (%)**						
pT1	28 (44.4)	176 (49.0)	0.736	9 (32.1)	25 (34.2)	1
pT2	35 (55.6)	179 (49.9)		18 (64.3)	45 (61.6)	
pT3	0 (0.0)	4 (1.1)		1 (3.6)	3 (4.1)	
NA	0	2		0	0	
**pN (%)**						
pN0	33 (52.4)	167 (46.4)	0.787	19 (86.4)	45 (78.9)	1
pN1	18 (28.6)	111 (30.8)		3 (13.6)	9 (15.8)	
pN2	7 (11.1)	55 (15.3)		0 (0.0)	2 (3.5)	
pN3	5 (7.9)	27 (7.5)		0 (0.0)	1 (1.8)	
NA	0	1		6	16	

Values that are not available (NA) for categorical variables are shown but were not included in the statistical analysis. Statistical tests of continuous variables were performed using the two-sided Kruskal–Wallis rank sum test. Statistical tests of categorical variables were performed using the two-sided Fisher’s exact test.

*IDC* invasive ductal carcinoma, *ILC* invasive lobular carcinoma, *IHC* immunohistochemistry, *HR* hormone receptor, *sTIL* stromal tumor-infiltrating lymphocyte, *iTIL* intratumoral tumor-infiltrating lymphocyte, *SD* standard deviation.

**Fig. 2:**
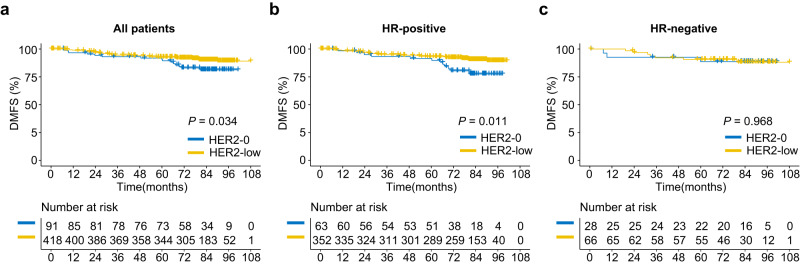
Difference in distant metastasis–free survival (DMFS) between HER2-low and HER2-0 patients. **a**–**c** Kaplan–Meier curves and risk tables showing DMFS of HER2-low and HER2-0 breast cancers compared by two-sided log-rank test in the entire cohort (**a**), HR-positive subgroup (**b**) and HR-negative subgroup (**c**). Source data are provided as a Source Data file.

We then investigated the difference in clinical outcome between HER2-low and HER2-0 breast cancer patients. The median follow-up times of all included patients and the HER2-low cohort were 6.9 years (interquartile range [IQR] 5.9–7.5 years) and 6.9 years (IQR 6.0–7.5 years), respectively. HER2-low patients showed a significantly better prognosis in terms of distant metastasis–free survival (DMFS) in the entire cohort (*P* = 0.034, log-rank test) and HR-positive patients (*P* = 0.011) but not in the HR-negative cohort (*P* = 0.968) (Fig. 2a–c). Regarding overall survival (OS), no significant difference was found between groups based on HR status (Supplementary Fig. S4a–c).

### Distinctness of HER2-low breast cancers with different HR statuses

Whether HER2-low breast cancer is distinct from other HER2-negative (i.e., HER2-0) tumors is disputed. Here, we present the results of multiomics analysis of our large-scale Chinese dataset.

**Fig. 3:**
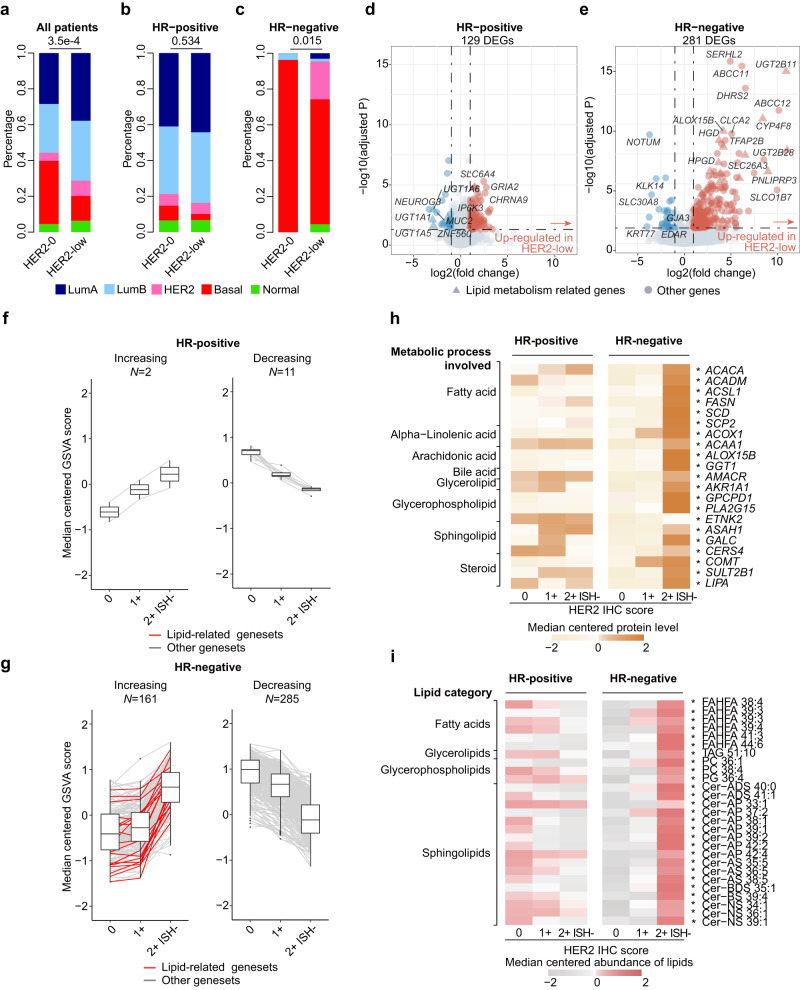
Difference in the molecular background between HER2-low and HER2-0 breast cancers with different HR statuses. **a**–**c** Bar plots comparing the distribution of PAM50 subtypes between HER2-low and HER2-0 breast cancers in the entire cohort (**a**), HR-positive subgroup (**b**) and HR-negative subgroup (**c**). *P* values were computed using the two-sided Fisher’s exact test. **d**, **e** Dot plots showing differentially expressed genes (DEGs) between HER2-low and HER2-0 tumors in the HR-positive (**d**) and HR-negative (**e**) subgroups. *P* values were computed using the two-sided Wald’s test and were adjusted for multiple testing using the false discovery rate method. Genes with abs(log_2_(fold change))>1 and adjusted *P* value <0.05 were considered DEGs (colored blue or red). Lipid metabolism-related genes are represented by triangles, and others are represented by circles. **f**, **g** Boxplots showing REACTOME gene set activity scores changing with HER2 IHC scores in the HR-positive (**f**) and HR-negative (**g**) subgroups. The number (*N*) of increasing or decreasing gene sets is indicated. The centerline represents the median, the box limits represent the upper and lower quartiles, the whiskers represent the 1.5× interquartile range, and the points represent individual samples. **h** Heatmap showing the protein levels of lipid metabolism-related genes across HER2 IHC scores in the HR-positive and HR-negative subgroups. Proteins marked with * represent those that increase significantly with HER2 IHC scores. **i** Heatmap showing the abundance of lipids across HER2 IHC scores in the HR-positive and HR-negative subgroups. Lipids marked with * represent lipids that increase significantly with HER2 IHC scores. LumA luminal A, LumB luminal B, HER2 (in PAM50 section) HER2-enriched, basal basal-like, normal normal-like, ISH in situ hybridization. Source data are provided as a Source Data file.

We first used some well-established molecular subtyping methods to profile the overall molecular background of HER2-low breast cancers. First, in terms of widely acknowledged transcriptome-based PAM50 intrinsic subtypes^14^, we found that compared with HER2-0 patients, HER2-low patients contained fewer basal-like tumors (35.2% vs. 13.8%, *P* = 3.5e–4, Fisher’s exact test) (Fig. 3a and Supplementary Table S1). After HR stratification, a significant difference existed only in the HR-negative subgroup (69.7% vs. 96.3%, *P* = 0.015, Fisher’s exact test) (Fig. 3b, c and Supplementary Table S2), implying that consequent analysis should be performed separately in HR-positive and HR-negative breast cancers. Similarly, following the IntClust subtyping system^15,16^, HR-negative HER2-low tumors included significantly fewer basal-like-enriched IntClust 10 subtypes than HER2-0 tumors (56.6% vs. 82.6%, *P* = 0.038, Fisher’s exact test) (Source Data). This discrepancy between HER2-low and HER2-0 breast cancers in the HR-negative subgroup was also implied by several specialized TNBC subtyping systems with the different assignments of molecular subtypes, including FUSCC-TNBC subtype^10^, Burstein’s subtype^17^, Lehman’s subtype^18^ and Quist’s TNBC subtype^19^ (Table 2).

**Table 2:** Molecular subtypes of HER2-low and HER2-0 breast cancers in TNBCs

	HER2-0	HER2-low	*P*
	*N* = 28	*N* = 73	
**FUSCC-TNBC subtype (%)**			
LAR	0 (0.0)	17 (25.8)	0.010
IM	10 (37.0)	19 (28.8)	
BLIS	15 (55.6)	27 (40.9)	
MES	2 (7.4)	3 (4.5)	
NA	1	7	
**Burstein’s subtype (%)**			
LAR	2 (7.4)	23 (34.8)	0.036
MES	4 (14.8)	6 (9.1)	
BLIA	6 (22.2)	12 (18.2)	
BLIS	15 (55.6)	25 (37.9)	
NA	1	7	
**Lehmann’s subtype (%)**			
LAR	0 (0.0)	9 (14.8)	0.068
UNS	3 (12.0)	5 (8.2)	
IM	9 (36.0)	20 (32.8)	
M	6 (24.0)	6 (9.8)	
BL2	0 (0.0)	7 (11.5)	
BL1	7 (28.0)	14 (23.0)	
NA	3	12	
**Quist’s subtype (%)**			
MC1	0 (0.0)	12 (18.2)	0.019
MC2	0 (0.0)	9 (13.6)	
MC3	4 (14.8)	6 (9.1)	
MC4	3 (11.1)	5 (7.6)	
MC5	3 (11.1)	7 (10.6)	
MC6	17 (63.0)	27 (40.9)	
NA	1	7	

Values that are not available (NA) for categorical variables are shown but were not included in the statistical analysis. Statistical tests were performed using Fisher’s exact test.

*TNBC* triple-negative breast cancer, *FUSCC* Fudan University Shanghai Cancer Center.

To better demonstrate the uniqueness of HR-negative HER2-low breast cancers, we next comprehensively compared the molecular differences between HER2-low and HER2-0 tumors stratified by HR status using multiomics data. In terms of differentially expressed genes (DEGs), in the HR-negative subgroup, more genes were differentially expressed, and the degree of difference was also more marked, especially for lipid metabolism-related genes (Fig. 3d, e). By performing enrichment analysis of these DEGs, we further verified active lipid metabolism in HR-negative HER2-low breast cancers (Supplementary Fig. S5a, b, Source Data), which was further validated at the proteome level (Supplementary Fig. S5c–f).

To obtain a deeper understanding of HER2-low breast cancers, we took HER2 IHC scores into consideration in the subsequent analysis. First, we investigated the change in the gene set enrichment scores along with the HER2 IHC scores in each HR subgroup. In the HR-negative subgroup, there were many more increasing and decreasing gene sets compared with the HR-positive subgroup at the transcriptomic level (161 vs. 2 for increasing, 285 vs. 11 for decreasing, respectively) (Fig. 3f, g). In accordance with the differential analysis above, lipid metabolism-related gene sets showed strong enrichment in the HR-negative subgroup (Fig. 3f, g, indicated by red lines) and were among the gene sets with the highest fold change (Supplementary Fig. S6a, b). The above findings were also validated at the proteomic and metabolomic levels separately. Specifically, in the HR-negative subgroup, the protein level of lipid metabolism genes (Fig. 3h) and the abundance of lipids (especially sphingolipids) increased along with the HER2 IHC scores (Fig. 3i and Supplementary Fig. S6c), which was consistent with the transcriptomic data.

### Distinct subgroups exist in Chinese patients with HR-negative HER2-low breast cancer

**Fig. 4:**
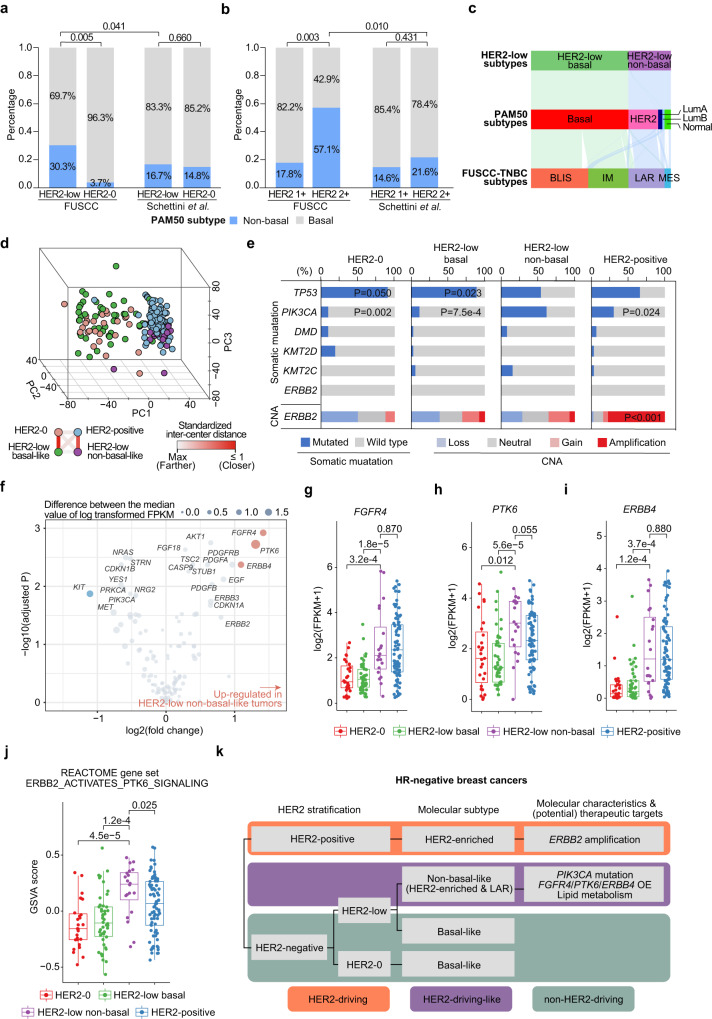
Interpatient molecular heterogeneity of HR-negative HER2-low breast cancers. **a**, **b** Bar plots comparing the proportion of non-basal-like tumors between the HER2-low and HER2-0 tumors (**a**) or between HER2 1+ and HER2 2+ ISH– tumors (**b**) in our FUSCC cohort and Schettini et al.’s cohort. *P* values were computed using the two-sided Fisher’s exact test. **c** Sankey diagram showing the classification of non-basal-like tumors in the PAM50 subtype and the FUSCC-TNBC subtype. **d** Principal component (PC) analysis of all protein-coding RNAs showing the relationship of HER2-0, HER2-low basal-like, HER2-low non-basal-like and HER2-positive tumors in HR-negative breast cancers. **e** Bar plots showing the somatic mutation rate of the top 5 genes within HR-negative breast cancers and genomic alterations of *ERBB2* across the HER2 subgroups. The *P* values of genes that showed significant differences between HER2-low non-basal-like tumors and other subgroups are annotated. *P* values were computed using the two-sided Fisher’s exact test. **f** Dot plots showing the differentially expressed genes (DEGs) involved in PI3K and *ERBB2* signaling between HER2-low non-basal-like and basal-like tumors. *P* values were computed using the two-sided Wilcoxon test and were adjusted for multiple testing using the false discovery rate method. Genes with abs_2_(log(fold change))>1 and adjusted *P* value < 0.05 were considered DEGs (colored blue or red). **g**–**i** Boxplot comparing the mRNA levels of *FGFR4* (**g**), *PTK6* (**h**) and *ERBB4* (**i**) among the HER2-0 (*N* = 27), HER2-low basal-like (*N* = 46), HER2-low non-basal-like (*N* = 20) and HER2-positive (*N* = 81) subgroups. *P* values were computed using the two-sided Wilcoxon test. The centerline represents the median, the box limits represent the upper and lower quartiles, the whiskers represent the 1.5× interquartile range, and the points represent individual samples. **j** Boxplots comparing the enrichment score of *REACTOME ERBB2 ACTIVATES PTK6 SIGNALING* among the HER2-0 (*N* = 27), HER2-low basal-like (*N* = 46), HER2-low non-basal-like (*N* = 20) and HER2-positive (*N* = 81) subgroups. *P* values were computed using the two-sided Wilcoxon test. The centerline represents the median, the box limits represent the upper and lower quartiles, the whiskers represent the 1.5× interquartile range, and the points represent individual samples. **k** Schematic diagram of the molecular characteristics and driving mechanisms of HR-negative HER2-low breast cancers. BLIS basal-like and immune-suppressed, IM immunomodulatory, LAR luminal androgen receptor, MES mesenchymal-like, OE overexpression. Source data are provided as a Source Data file.

As suggested above, up to 30.3% of HR-negative HER2-low patients had non-basal-like disease, which was significantly higher than that among HR-negative HER2-0 patients in our cohort (30.3% vs. 3.7%, *P* = 0.005, Fisher’s exact test) (Fig. 4a). This proportion was also significantly higher than that in Western HR-negative HER2-low cohorts^9^ (30.3% vs. 16.7%, *P* = 0.041, Fisher’s exact test) (Fig. 4a). When we subdivided HER2-low breast cancers into HER2 1+ and HER2 2+ ISH– tumors, we found a higher proportion of non-basal-like tumors in the HER2 2+ subgroup than in the HER2 1+ subgroup (57.1% vs. 17.8%, *P* = 0.003, Fisher’s exact test) (Fig. 4b). This discrepancy also corresponded to our findings in the molecular landscape above (Fig. 1e). In addition, the proportion of non-basal-like tumors in the HER2 IHC 2+ subgroup in our cohort was more than twice as high as that in the Western population^9^ (57.1% vs. 21.6%, *P* = 0.010, Fisher’s exact test) (Fig. 4b), which further indicated the interpatient heterogeneity among Chinese patients with HER2-low breast cancer.

Then, we further investigated the interpatient heterogeneity of HER2-low breast cancers. As shown in Fig. 1e, compared with the HER2 IHC score, the interpatient heterogeneity of HR-negative HER2-low breast cancer was more closely correlated with the PAM50 subtype in terms of basal-like tumors vs. non-basal-like tumors. Non-basal-like tumors were primarily characterized as the HER2-enriched subtype by the PAM50 system (14/20, 70.0%) (Fig. 4c). These tumors were robustly enriched in the LAR subtype in multiple TNBC subtyping systems^10,17,18^ (Fig. 4c and Supplementary Fig. S7a, b), which has been reported to be enriched in Chinese patients with TNBCs^10^. In Quist’s subtyping, non-basal-like tumors almost solely consisted of the MC1 and MC2 subtypes (Supplementary Fig. S7c), which appeared only in the HER2-low group and not in the HER2-0 group (Table 2) and were both exclusively immunomodulator negative^19^. In the principal component analysis of all genes, HER2-low non-basal-like tumors also greatly differed from basal-like tumors (Fig. 4d). Specifically, HER2-low basal-like tumors and HER2-0 tumors were highly similar, while HER2-low non-basal-like tumors were more similar to HER2-positive tumors, which emphasized the uniqueness of HER2-low non-basal-like tumors from the rest of TNBCs.

In these unique HR-negative HER2-low non-basal-like tumors, we further investigated their molecular features, driving mechanisms and therapeutic targets. Although these tumors mostly consisted of HER2-enriched tumors and mimicked HER2-positive breast cancers, no significant overexpression, gain/amplification or elevated mutation rate was discovered for *ERBB2* (Fig. 4e and Supplementary Fig. S8a, b). We further assessed other potential driving mechanisms of these tumors, and a high frequency of *PIK3CA* mutation (61.5%) was observed; the frequency was significantly higher than that in HER2-low basal-like, HER2-0 and HER2-positive tumors (10.8%, 9.1% and 27.3%, respectively, all *P* < 0.05, Fisher’s exact test) (Fig. 4e). Using gene set variation analysis (GSVA), we found an upregulated enrichment score for PI3K-AKT pathway activation (Supplementary Fig. S8c) and identified the potential role of PI3K activation in *ERBB2* signaling (Supplementary Fig. S8d) in these non-basal-like tumors. However, there were no significant differences between *PIK3CA* wild-type and mutated samples within the non-basal-like population in either pathway (*P* = 0.354 and *P* = 0.833, respectively, Wilcoxon test) (Supplementary Fig. S8e, f), indicating that there might be other pivotal mechanisms supporting these tumors in addition to *PIK3CA* mutation. Thus, we inspected all genes involved in PI3K and *ERBB2* signaling (Fig. 4f). We found that *FGFR4*, *PKT6* and *ERBB4* were significantly overexpressed in HER2-low non-basal-like tumors at the transcriptome level (Fig. 4g–i), which was supported by proteome data (Supplementary Fig. S8g) and further validated in external cohorts (Supplementary Fig. S8h, i). For these three genes, no difference in CNAs was observed between non-basal-like and basal-like tumors (Supplementary Fig. S7j–l), indicating that the difference was not driven by CNAs. For *PTK6*, we also observed elevated gene set scores for its role in *ERBB2* signaling (Fig. 4j), further emphasizing its importance. Notably, the expression levels of *PTK6*, *FGFR4* and *ERBB4* were not correlated (Supplementary Fig. S8m–o), suggesting their independent role in non-basal-like tumors.

In addition to the potential driving events, we comprehensively analyzed the molecular characteristics of HR-negative HER2-low non-basal-like tumors. We observed the activation of multiple metabolic processes, including lipid metabolism (Supplementary Fig. S8p, q). Furthermore, we demonstrated the correlation between *PIK3CA* mutation, *FGFR4/PTK6/ERBB4* overexpression and lipid metabolism in this subgroup of patients (Supplementary Fig. S9). The correlations indicated the potential interactions among these molecular features, which might jointly contribute to a HER2-drving-like landscape for HR-negative HER2-low non-basal-like tumors (Fig. 4k).

### Alterations in 17q were correlated with improved prognosis in HR-positive HER2-low breast cancer patients

The DMFS of HER2-low breast cancer patients was significantly improved compared with that of HER2-0 patients in the HR-positive subgroup (Fig. 2b). As indicated by previous studies^9,20^, the superior prognosis of HER2-low breast cancer might result from the higher expression of luminal-related genes^9^, which might improve their response to endocrine therapy. However, in our dataset, we did not observe a significant difference in the expression of luminal-related genes (except for higher *BCL2* expression in HER2-low tumors, *P* = 0.050, Wilcoxon test) (Supplementary Fig. S10a) or higher endocrine sensitivity scores^21,22^ (Supplementary Fig. S10b, c) in HR-positive HER2-low tumors.

As shown above, the discrepancy between HER2-low and HER2-0 tumors of HR-positive status was relatively small at the transcriptomic, proteomic and metabolomic levels. Thus, we mainly focused on genomic alterations. In HR-positive HER2-low diseases, the most frequently mutated cancer-associated genes (CAGs)^23^ were *PIK3CA* (45%), *TP53* (24%), *GATA3* (16%) and *MAP3K1* (13%) (Supplementary Fig. S10d). However, no CAGs were found to be differentially mutated compared with HER2-0 tumors.

**Fig. 5:**
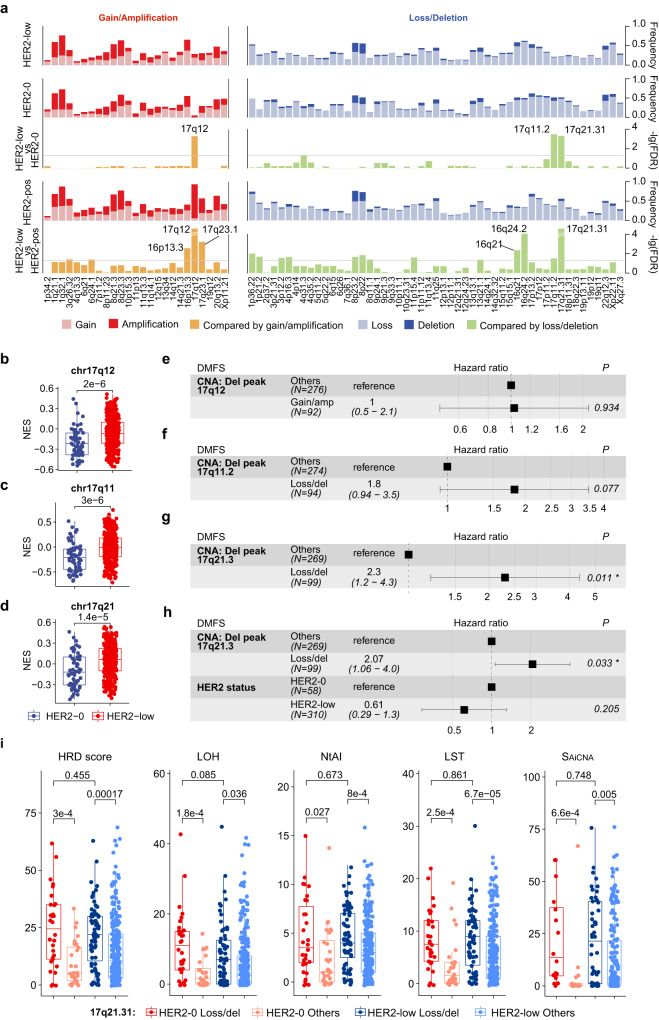
Relation between molecular alterations and survival in HR-positive HER2-low breast cancer. **a** Bar plots showing somatic copy number alteration (CNA) frequency and –log10(FDR) for comparison using an FDR-adjusted two-sided Fisher’s exact test between the HER2 status subgroups. The gray horizontal line in the comparison plots represents the level of –log10(0.05). **b**–**d** Gene set variation analysis (GSVA) scores of chr17q12 (**b**), chr17q11 (**c**) and chr17q21 (**d**) using the Molecular Signatures Database (MSigDB) C1 collection between HER2-0 (*N* = 61) and HER2-low (*N* = 355) breast cancers. *P* values were computed using a two-sided Fisher’s exact test. The centerline represents the median, the box limits represent the upper and lower quartiles, the whiskers represent the 1.5× interquartile range, and the points represent individual samples. **e**–**g** Forest plots showing the univariable Cox regression analysis for distant metastasis–free survival (DMFS) of the status of focal peaks 17q12 (**e**), 17q11.2 (**f**) and 17q21.31 (**g**) in HR-positive HER2-low breast cancers. Number (*N*) of patients belonging to each category is indicated. The association of all variables with prognosis was analyzed using a two-sided Cox proportional hazard regression analysis. Error bars represent the 95% confidence interval of the hazard ratio. **h** Forest plots showing the multivariable Cox regression analysis for distant metastasis–free survival (DMFS) of the status HR and the status of focal peak 17q21.31 in HR-positive HER2-low breast cancers. Number (*N*) of patients belonging to each category is indicated. The association of all variables with prognosis was analyzed using a two-sided Cox proportional hazard regression analysis. Error bars represent the 95% confidence interval of the hazard ratio. **i** Boxplots showing the genomic instability index related to 17q21.31 loss/deletion. The number (*N*) of HER2-0 loss/deletion, HER2-0 others, HER2-low loss/deletion and HER2-low others is 30, 28, 71 and 248. *P* values were computed using the two-sided Wilcoxon test. In boxplots, the centerline represents the median, the box limits represent the upper and lower quartiles, the whiskers represent the 1.5× interquartile range, and the points represent individual samples. Gain/amp gain/amplification, Loss/del loss/deletion. Source data are provided as a Source Data file.

Differences in the CNAs of several focal peaks were found: HER2-low tumors showed significantly higher levels of 17q12 gain/amplification (28.5% vs. 5.2%, false discovery rate (FDR) <0.001, mainly attributed to gains) and lower levels of 17q11.2 loss/deletion (21.3% vs. 51.7%, FDR <0.001) and 17q21.31 loss/deletion (22.3% vs. 51.7%, FDR <0.001) than HER2-0 tumors (Fig. 5a), which was also validated in the TCGA-BRCA (The Cancer Genome Atlas Breast Invasive Carcinoma) cohort (Supplementary Fig. S10e–g). To further evaluate the downstream effect of these CNAs, we performed GSVA using MSigDB C1 collections (positional gene sets), and significantly higher enrichment scores were observed in HER2-low tumors (Fig. 5b–d).

In univariate Cox regression analysis, gain/amplification in 17q12 was not correlated with DMFS (hazard ratio = 1, *P* = 0.934) (Fig. 5e). However, for 17q11.2, its loss/deletion status was related to worse DMFS with borderline significance (hazard ratio = 1.8, *P* = 0.077) (Fig. 5f). The loss/deletion of 17q21.31 was significantly correlated with worse DMFS (hazard ratio = 2.3, *P* = 0.011) (Fig. 5g). In the following multivariate Cox analysis, the loss/deletion of 17q21.31 and 17q11.2 was also correlated with worse DMFS after correcting for HER2 status (17q21.31: hazard ratio = 2.07, *P* = 0.003; 17q11.2: hazard ratio = 1.59, *P* = 0.191) (Fig. 5h and Supplementary Fig. S10h, i).

We then investigated the potential mechanisms underlying the relationship between clinical outcome and alterations in 17q21.31 and 17q11.2. We found that the copy numbers 17q21.31 and 17q11.2 were highly correlated with the copy number 17q12 (Supplementary Fig. S10j), although there was little amplification in 17q12. We then focused on specific genes in 17q21.31 and 17q11.2. At the RNA level, most of the genes in these two focal peaks showed higher expression levels in non-loss/deletion tumors (Supplementary Fig. S11a, Source Data). The difference was also confirmed at the protein level (Supplementary Fig. S11b, c), where *NF1* in 17q11.2 and *NBR1* in 17q21.31 were among the most upregulated genes at both the RNA and protein levels, respectively. In addition, considering that *BRCA1* was in 17q21.31, we investigated the difference in genomic instability scores. The effect of 17q21.31 loss/deletion was further implied by the increase in multiple chromosomal instability markers, including the homologous recombination deficiency (HRD) score^24,25^, large-scale state transitions (LSTs)^26^, number of telomeric allelic imbalance events (NtAI)^27^, loss of heterozygosity (LOH)^28^ and allelically imbalanced CNAs (AiCNAs)^29^, in these tumors (Fig. 5i).

## Discussion

HER2-low breast cancer is an emerging subtype that includes over half of all breast cancer patients. Here, we established a multiomics HER2-low breast cancer cohort in Chinese (*N* = 434) and comprehensively revealed the molecular nature, interpatient heterogeneity, and racial and ethnic differences among HER2-low breast cancers. We further proved the pivotal role of HR stratification^12,20^ and further characterized HER2-low breast cancers more profoundly among TNBCs and luminal breast cancers. Our work may provide evidence for more precise management of HER2-low breast cancers in the future.

Our HER2-low breast cancer study is a significant supplement to current studies on HER2-low breast cancer. To date, most studies in this field have characterized HER2-low breast cancer utilizing clinicopathological and prognostic information^6,20,30^, lacking a comprehensive view from a molecular perspective. Although some studies have tried to introduce molecular information, they were typically focused on one type of omics analysis^7,9,12^. Studies based on TCGA dataset might also be impacted by outdated criteria for IHC-based HER2 subtyping^13^, which may not be suitable for current research on HER2-low breast cancer. Our study provides a multiomics HER2-low breast cancer dataset with reliable rescored HER2-low classification data and WES, CNA assay, transcriptome, proteome, and metabolome data. In addition, our cohort was a large-scale multiomic HER2-low breast cancer cohort in a Chinese population, and our analysis suggests potential ethnic and race disparities in HER2-low breast cancers, which further emphasized its significance.

According to our cohort, HR-negative HER2-low breast cancers might represent a distinct molecular entity in Chinese patients with TNBC in terms of its special features in terms of molecular subtype, somatic mutations, gene expression and metabolic characteristics from HER2-0 tumors. Interpatient heterogeneity was characterized within HR-negative HER2-low breast cancers by identifying two distinct subgroups, and we found that the distinctness of HER2-low breast cancers might result from the enrichment of the distinct non-basal-like tumors. Specifically, basal-like tumors mimicked HER2-0 breast cancers; non-basal-like tumors (primarily the HER2-enriched, LAR and MC1/MC2 subtypes) were similar to HER2-positive breast cancers and were enriched in Chinese. Non-basal-like tumors showed higher HER2 expression and activation of the PI3K-AKT pathway, which might indicate greater opportunities to benefit from T-DXd according to preclinical studies^31,32^. In addition, non-basal-like tumors featured a high frequency of *PIK3CA* mutation and overexpression of *FGFR4*/*PTK6/ERBB4*, providing potential targets and coadministration choices with ADCs for these patients. Mechanistically, *FGFR4* is a PAM50 gene, and its overexpression is a characteristic of the HER2-enriched subtype^14^, possibly giving rise to the enrichment of the HER2-enriched subtype. *ERBB4* can heterodimerize with *ERBB2* to activate downstream pathways^33^. *PTK6* and *PIK3CA* can function as downstream effectors of *ERBB2* signaling^34–36^. These factors might jointly contribute to the HER2-drving-like molecular landscape of non-basal-like HER2-low breast cancers. In addition, we revealed the unique lipid metabolism features of these non-basal-like tumors, which revealed potential future treatment strategies such as targeting ferroptosis^37^. The results of the subgroup analysis from the DESTINY-Breast04 trial informed us about the potential difference in T-DXd response between Asian participants and white participants^8^. Our results further revealed the potential racial and ethnic differences in HER2-low patients at the molecular level, which may deepen clinicians’ understanding of these patients and promote more precise management.

For HR-positive tumors, HER2-low breast cancers showed relatively minor differences from HER2-0 tumors at the molecular level. Nevertheless, among HR-positive patients, HER2-low patients had significantly better DMFS outcomes than HER2-0 breast cancer patients, in concordance with some previous studies^20,30,38–40^ (different times to events were used in these studies). We found lower levels of 17q21.31 and 17q11.2 loss/deletion in HER2-low breast cancers than in HER2-0 tumors, which was correlated with a better prognosis in luminal patients. The high correlation among the copy numbers of 17q21.31, 17q11.2 and 17q12 might reflect the 17q12-21 locus (or 17q12-21 amplicon)^41,42^ or chromothripsis of 17q in ER-positive breast cancers^43,44^ despite the lack of high amplification in 17q12. Genes located on these peaks, such as *NF1*^45^, *NBR1*^46^ and *BRCA1*^47,48^, were also considered related to tumor biology and patient survival. Larger-scale prospective studies and investigations into molecular mechanisms are needed to further reveal the relationship between these CNAs and clinical outcomes.

There are some limitations to our study. First, our study was based on a single-center retrospective cohort, and larger-scale prospective studies need to be carried out to validate our findings before clinical application. Second, while we carefully designed and performed the rescoring of all HER2-negative breast cancers to minimize potential bias resulting from interobserver differences and changes in diagnosis guidelines, the accurate discrimination of specific IHC scores is still challenging, especially in the low range (IHC 0 or 1+)^49^. More precise or specialized diagnostic systems are urgently needed.

Our work provides a comprehensive multiomics HER2-low breast cancer dataset and suggests that the distinct nature, interpatient heterogeneity, and racial and ethnic differences of HER2-low breast cancer are dependent on HR status, and these features were more marked in the HR-negative subgroup. We also revealed a potential driving mechanism and proposed possible therapeutic targets for some HER2-low breast cancers. These findings might help to stratify HER2-low patients more precisely in terms of HR status, molecular subtypes and race and ancestry information for future studies and practices in the era of ADCs.

## Methods

### Cohort design and clinical characteristics

This study was approved and supervised by the Institutional Review Board of FUSCC (IRB ID: 050432-4-1911D). All enrolled patients were fully informed of their rights and signed written consent forms. Chinese patients nationwide diagnosed with breast cancer who were treated at the Department of Breast Surgery at FUSCC from January 1, 2013, to December 31, 2014, were retrospectively reviewed. A total of 773 consecutive patients were enrolled according to the following defined criteria: (1) willingness to participate in this study and signed written informed consent; (2) diagnosis of unilateral invasive breast cancer; (3) central pathologic examination of tumor specimens performed by the Department of Pathology at FUSCC: ER, PR, and HER2 statuses; (4) no evidence of distant metastasis at diagnosis; and (5) sufficient archived tissue available for further investigation.

Demographic variables of participants, including age, investigator-observed sex and menopause status, were collected. Due to the sex-tendency of breast cancer, only one male was included in this study, and thus sex gender analysis was not carried out. The primary tumor samples were collected during surgery and underwent pathology examination and multiomics profiling. Pathology examination of ER, PR, HER2 and Ki67 was performed by the Department of Pathology of FUSCC. ER/PR negativity was defined as <1% positively stained cells in IHC testing. All pathology results were confirmed independently by two experienced pathologists according to the latest American Society of Clinical Oncology/College of American Pathologists guidelines at the time. Tumor-infiltrating lymphocytes were manually assessed according to the recommendations by the International TILs Working Group^50^. The disease stage was assessed according to the latest TNM classification edition of the American Joint Committee on Cancer. OS was defined as the time from the initial surgery to the date of death due to any cause or the last follow-up date. DMFS was defined as the time from the initial surgery to the date of the first distant metastasis diagnosis or death due to any cause.

### Tumor sample profiling

Collected tumor samples were preserved in the biobank of FUSCC. Tumors underwent WES (49122 somatic mutations were identified), OncoScan CNA assay (27100 somatic gene-level CNAs and 76 focal peaks were identified), RNA sequencing (19,892 mRNAs were identified), TMT quantitative proteomics (7952 proteins, 153,441 unique peptides, and 3,430,404 peptide-spectrum matches were identified) and metabolomics (669 polar metabolites and 1312 lipids were identified) to generate multiomics data. Detailed information regarding the sample processing and multiomics information generation is provided in the Supplementary Methods.

### Evaluation and rescoring of HER2 status

We utilized the Ventana BenchMark Ultra automatic stainer and the Ventana Ultra View universal DAB detection kit (both from Ventana Medical System Inc., Roche Tucson, Arizona, USA) to perform IHC staining of HER2. We exclusively employed antibodies sourced from Roche Ventana. Subsequently, a dual-probe fluorescence in situ hybridization (FISH) test was performed for those samples with equivocal IHC results using the PathVysion HER2 DNA probe Kit (Vysis Inc. in Downers Grove, IL) on the same specimen as the IHC test. The IHC and FISH results were independently interpreted by two highly experienced pathologists.

For rescoring, we first checked the quality of historical HER2 IHC slides after storage. We inspected the staining status of the historical slides, with external slide controls for IHC (via tissue microarray (TMA) core samples) as a reference. The results showed that the historical HER2 IHC slides were well preserved without significant shading. Then, historical HER2 IHC slides were scored independently by three experienced pathologists (W.Y., M.L., X.X.) of the Department of Pathology of FUSCC, who received specialized training previously provided by the diagnostic reagent supplier. The discordantly scored samples were finally scored in a consensus session. All pathologists were blinded to the original IHC scores and ISH results and were informed to discriminate each HER2 IHC score accurately. All evaluations were performed according to the 2018 ASCO/CAP guidelines^11^. HER2-0 was defined as a HER2 IHC score of 0; HER2-low was defined as a HER2 IHC score of 1+ or 2+ with ISH–.

### Statistical analysis

Comparisons of ordered class variables and continuous variables were performed by the Kruskal–Wallis rank sum test. Comparisons of categorical variables were performed by Fisher’s exact test or the *χ*^2^ test, where appropriate. All statistical tests were two-sided, and specific methods were annotated in addition to the *P* value in the text. FDRs were obtained using the *p.adjust* function in R with the “fdr” method. Survival analysis was performed using the Kaplan–Meier method and compared with the log-rank test using the R package “survival” (version 3.3-1) and then visualized by the R package “survminer” (version 0.4.9). All analyses above were performed in R version 4.0.4.

### Multiomics analysis

Comparison of CNAs in “threshold” form was performed by Fisher’s exact test for gain/amplification and loss/deletion separately. The *P* value was adjusted using the FDR method. DEGs were identified using the R package “DESeq2” (version 1.30.1) according to official instructions^51^. Differentially expressed proteins were determined using the Wilcoxon test. Enrichment analysis based on DEGs and differentially expressed proteins was performed using the R package “clusterProfiler” (version 4.0.5). Lipid metabolism genes were extracted from the Kyoto Encyclopedia of Genes and Genomes (KEGG) database (https://www.kegg.jp/). Gene lists of PI3K and *ERBB2* signaling and REACTOME gene sets were derived from the *MSigDB* C2 collection (www.gsea-msigdb.org)^52,53^. DEGs in those genes between HER2-low non-basal-like and basal-like tumors were determined using the Wilcoxon test. Genes with abs(log_2_(fold change))>1 and FDR <0.05 were considered significant. The change of REACTOME gene set enrichment scores along with the HER2 IHC scores was evaluated by the combination of the Kruskal–Wallis test and Spearman’s correlation analysis across HER2 IHC scores using median centered log_2_(FPKM+1). Gene sets with Spearman’s rho >0.2 and Spearman’s FDR <0.25 were increasing, and those with Spearman’s rho <−0.2 and Spearman’s FDR <0.25 were decreasing. The changes of protein and metabolite levels along with the HER2 IHC scores were evaluated using a similar method described above. Proteins and metabolites (polar metabolites and lipids) with Spearman’s rho >0.3 and Spearman’s FDR <0.5 were increasing, and those with Spearman’s rho <−0.3 and Spearman’s FDR <0.5 were decreasing. Pathway-based analysis (DA score) was performed using lipids and polar metabolites that change along with HER2 IHC scores (Spearman’s rho >0.3 or Spearman’s rho <−0.3) as previously described^54^. Specifically, the DA score was defined as follows:

$${{{{{{\rm{DA}}}}}}}=\frac{{{{{{{\rm{No}}}}}}}.{{{{{{\rm{of}}}}}}\; {{{{{\rm{metabolites}}}}}}\; {{{{{\rm{increased}}}}}}}-{{{{{{\rm{No}}}}}}}.{{{{{{\rm{of}}}}}}\; {{{{{\rm{metabolites}}}}}}\; {{{{{\rm{decreased}}}}}}}}{{{{{{{\rm{No}}}}}}}.{{{{{{\rm{of}}}}}}\; {{{{{\rm{measured}}}}}}\; {{{{{\rm{metabolites}}}}}}\; {{{{{\rm{in}}}}}}\; {{{{{\rm{a}}}}}}\; {{{{{\rm{certain}}}}}}\; {{{{{\rm{pathway}}}}}}}}$$

In the molecular landscape of HER2-low breast cancers displayed in Fig. 1e, the top 10 CAGs^23^ with the highest mutation rate in the total HER2-low breast cancer cohort and CAGs that were differentially mutated between HER2 1+ and HER2 2+ tumors in each HR status subgroup were plotted. For CNA analysis, genes with the top 5 amplification rates and top 5 deletion rates among somatic CNAs in breast cancers, according to previous reports, were plotted^13,55^. For the transcriptome and proteome, the top 50 genes with the lowest FDR that were upregulated (top subblock) or downregulated (upper-middle subblock) in HR-positive HER2 1+ vs. HR-positive HER2 2+ and upregulated (lower-middle subblock) or downregulated (bottom subblock) in HR-negative HER2 1+ vs. HR-negative HER2 2+ were plotted. Significant genes (FDR <0.25) that were also included in tiers 1 and 2 of the Cancer Genomics Consortium (CGC) dataset (https://cancer.sanger.ac.uk/cosmic) were annotated. For lipids and polar metabolites in Supplementary Fig. S3, the top 25 metabolites with the lowest FDR that were upregulated (top subblock) or downregulated (upper-middle subblock) in HR-positive HER2 1+ vs. HR-positive HER2 2+ and upregulated (lower-middle subblock) or downregulated (bottom subblock) in HR-negative HER2 1+ vs. HR-negative HER2 2+ were plotted.

The gene set enrichment score for each sample was evaluated using the “gsva” function in the R package “GSVA” (version 1.38.2) according to package instructions^56^. Tumor mutation burden (TMB) was calculated with the R package “maftools” (version 2.6.05)^57^. PAM50 subtypes were determined based on the PAM50 classifier^14,58^. FUSCC-TNBC mRNA subtypes were determined according to our previous work^10^. Lehmann’s TNBC subtyping^18^ and Burstein’s TNBC subtyping^17^ were determined according to previous reports. IntClust subtypes were assigned using CNA and RNA data according to previous publications with R package iC10 (version 1.5)^15,16^. The endocrine sensitivity score^21^ and SET_ER/PR_^22^ were calculated according to previous studies. Genomic scar indexes, including HRD score^24,25^, LST^26^, NtAI^27^, LOH^28^ and AiCNA^29^, were calculated according to methods described in corresponding publications.

### Reporting summary

Further information on research design is available in the Nature Portfolio Reporting Summary linked to this article.

### Supplementary information

Supplementary Information

Reporting Summary

### Source data

Source Data

## Data Availability

Database for data collection: the hg38 human genome reference was downloaded from https://genome-idx.s3.amazonaws.com/hisat/grch38_snptran.tar.gz/. Cancer driver genes were derived from OncoKB (https://www.oncokb.org/), Integrative Onco Genomics (https://www.intogen.org/) and Cancer Genomics Consortium (CGC, https://cancer.sanger.ac.uk/cosmic/) dataset. Human protein database was downloaded from Universal Protein (Uniprot, https://www.uniprot.org/). Annotations for lipids were downloaded from LipidBlast database (https://fiehnlab.ucdavis.edu/projects/lipidblast/). Database for data analysis: the multiomics data and clinical information of the TCGA-BRCA dataset were downloaded from cBioPortal (https://www.cbioportal.org/). Lipid metabolism genes were extracted from the Kyoto Encyclopedia of Genes and Genomes (KEGG) database (https://www.kegg.jp/). Gene lists of PI3K and ERBB2 signaling and REACTOME gene sets were derived from the MSigDB C2 collection (https://www.gsea-msigdb.org/). The multiomics data and clinical information of the TCGA-BRCA dataset used in this study are available in the cBioPortal (https://www.cbioportal.org/)^59,60^. Data generated in this study: the WES data, CNA data, RNA sequencing data and metabolome data generated in this study have been deposited in the GSA database (https://ngdc.cncb.ac.cn/gsa/) and can be access by searching for biobroject PRJCA017539. The TMT-based MS-quantified protein data have been deposited in the iProX (https://www.iprox.cn) under accession codes IPX0006535000. A minimum dataset this study has been deposited in the Zenodo (https://zenodo.org/) under 10.5281/zenodo.8103633 [10.5281/zenodo.8103633]. The data are publicly accessible. For any further questions, please contact Zhi-Ming Shao (zhimingshao@fudan.edu.cn). We will respond in 10 business days. Source Data are provided with this paper.

## Code availability

The code is available at GitHub^61^ (https://github.com/ljdai98/FUSCC_HER2-low_breast_cancer_study; 10.5281/zenodo.7896926).
