# Supplementary material for: Molecular features and clinical implications of the heterogeneity in Chinese patients with HER2-low breast cancer
Source: Nat Commun. 2023 Aug 22;14:5112. doi: 10.1038/s41467-023-40715-x (PMC10444861; doi:10.1038/s41467-023-40715-x)
Supplement: Supplementary file 1 — Supplementary Information [file 41467_2023_40715_MOESM1_ESM.pdf]

Supplementary Information for

Molecular features and clinical implications of the heterogeneity in Chinese patients with HER2-low breast cancer

Lei-Jie Dai, Ding Ma, Yu-Zheng Xu, Ming Li, et al.

This file includes:

Supplementary Figures 1-11

Supplementary Table 1-2

Supplementary Methods

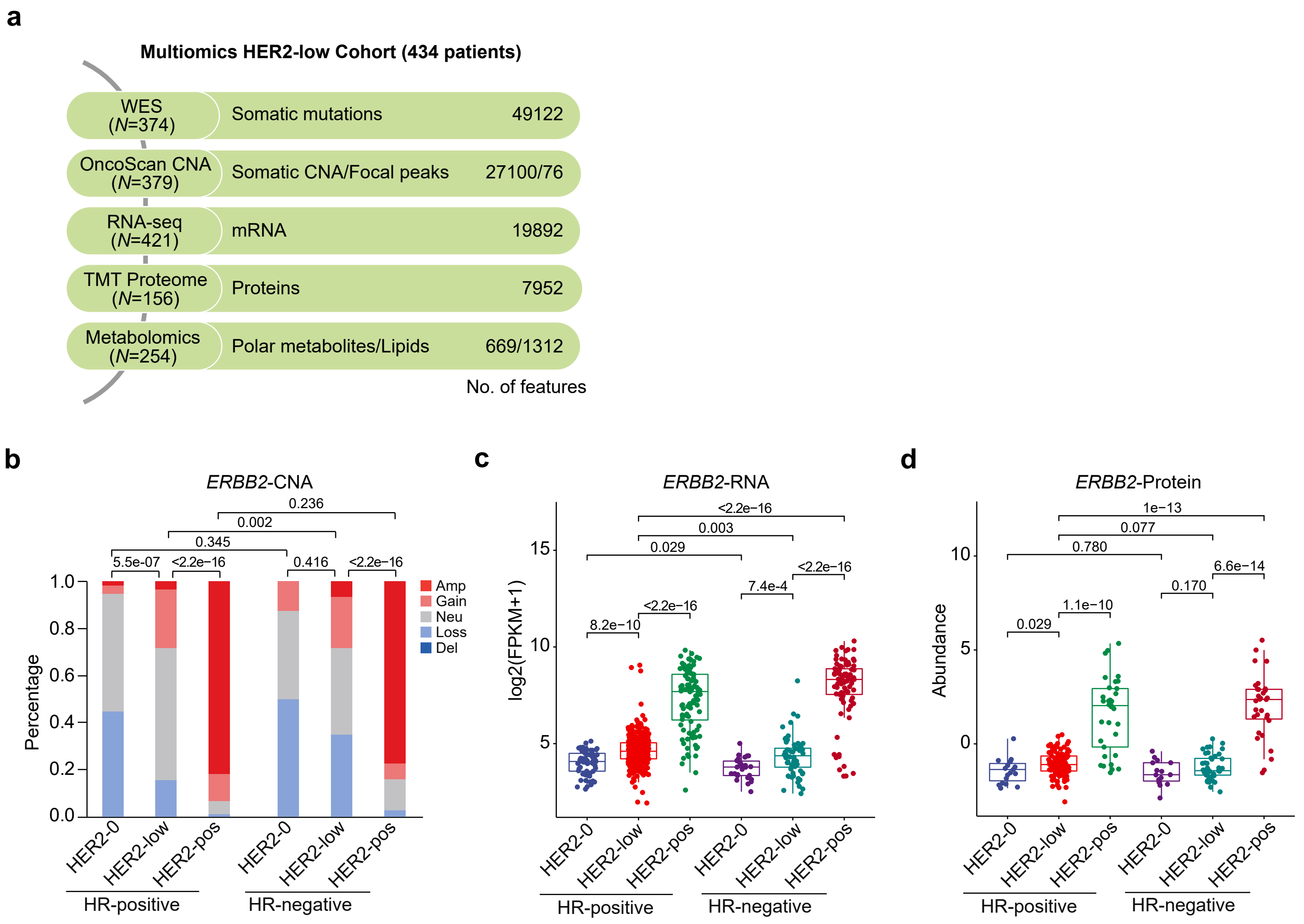
a
Multiomics HER2-low Cohort (434 patients)
WES (N=374)
Somatic mutations
49122
OncoScan CNA (N=379)
Somatic CNA/Focal peaks
27100/76
RNA-seq (N=421)
mRNA
19892
TMT Proteome (N=156)
Proteins
7952
Metabolomics (N=254)
Polar metabolites/Lipids
669/1312
No. of features
b
ERBB2-CNA
Percentage
Amp
Gain
Neu
Loss
Del
HER2-0
HER2-low
HER2-pos
HR-positive
HR-negative
c
ERBB2-RNA
log2(FPKM+1)
d
ERBB2-Protein
Abundance

Figure S1. The omics overview of the Fudan University Shanghai Cancer Center (FUSCC) HER2-low cohort, related to Figure 1.

(a) Multiomics level and number of features of included HER2-low patients. WES, whole exome sequencing; TMT: tandem mass tag.

(b) Bar plots comparing the copy number alteration (CNA) of *ERBB2* among HER2 status subgroups stratified by hormone receptor (HR) status based on Genomic Identification of Significant Targets in Cancer (GISTIC) analysis. Amp: 2, gain: 1, neu: 0, loss: -1, del: -2. P values were computed using the two-sided Fisher's exact test.

(c-d) Boxplots comparing the RNA (c) and protein (d) levels of *ERBB2* among HER2 status subgroups stratified by HR status. For RNA, the number (N) of HR-positive HER2-0, HR-positive HER2-low, HR-positive HER2-positive, HR-negative HER2-0, HR-negative HER2-low, and HR-negative HER2-positive is 61, 355, 100,27, 66, and 81. For protein, the number (N) was 19, 113, 33, 15, 43 and 31. P values were computed using the two-sided Wilcoxon test. In boxplots, the centerline represents the median, the box limits represent the upper and lower quartiles, the whiskers represent the 1.5x interquartile range, and the points represent individual samples.

Source data are provided as a Source Data file.

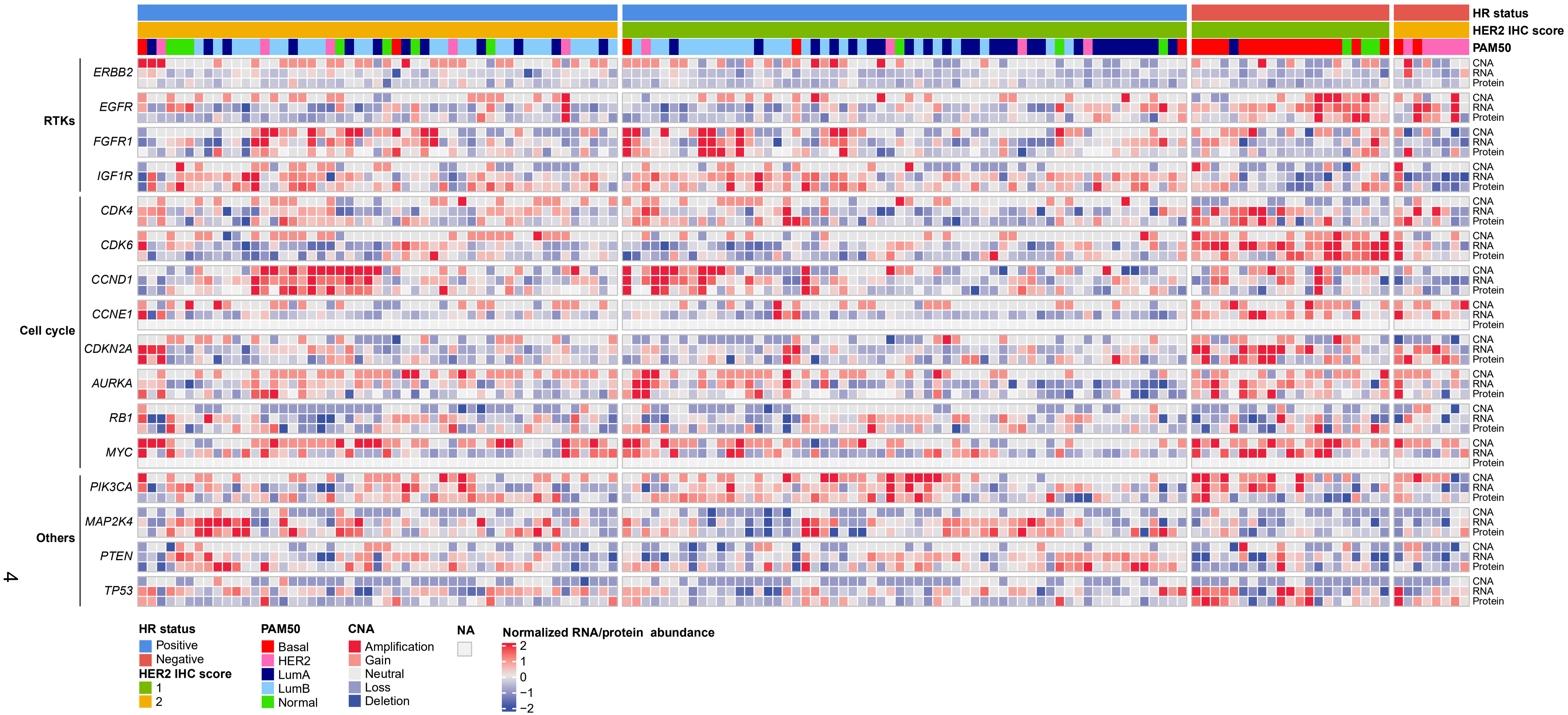

HR status
HER2 IHC score
PAM50
RTKs
ERBB2
EGFR
FGFR1
IGF1R
Cell cycle
CDK4
CDK6
CCND1
CCNE1
CDKN2A
AURKA
RB1
MYC
Others
PIK3CA
MAP2K4
PTEN
TP53
CNA
RNA
Protein
HR status
Positive
Negative
HER2 IHC score
1
2
PAM50
Basal
HER2
LumA
LumB
Normal
CNA
Amplification
Gain
Neutral
Loss
Deletion
NA
Normalized RNA/protein abundance
2
1
0
−1
−2

Figure S2. CNA-RNA-protein integrated analyses of frequently altered breast cancer genes in HER2-low breast cancers, related to Figure 1e.

Samples with all CNA, RNA and protein data were included. RTKs: receptor tyrosine kinases.

Source data are provided as a Source Data file.

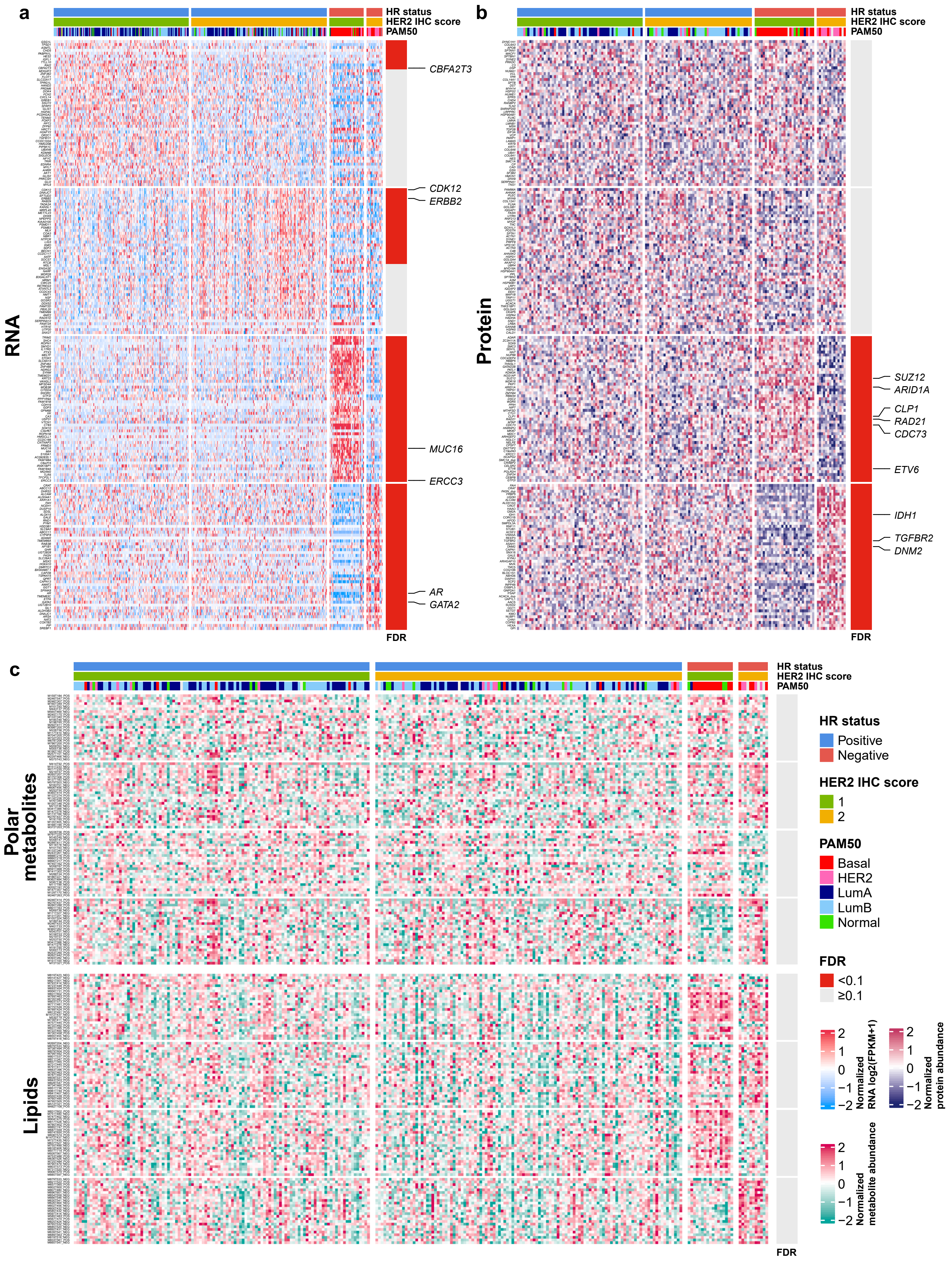
a
b
c
RNA
Protein
Polar metabolites
Lipids
HR status
HER2 IHC score
PAM50
FDR
CBFA2T3
CDK12
ERBB2
MUC16
ERCC3
AR
GATA2
SUZ12
ARID1A
CLP1
RAD21
CDC73
ETV6
IDH1
TGFBR2
DNM2
HR status
Positive
Negative
HER2 IHC score
1
2
PAM50
Basal
HER2
LumA
LumB
Normal
FDR
<0.1
≥0.1
Normalized RNA log2(FPKM+1)
Normalized protein abundance
Normalized metabolite abundance

Figure S3. Heatmap showing the molecular landscape of HER2-low breast cancers, related to Figure 1e.

(a-c) Samples were stratified by hormone receptor (HR) status and HER2 IHC scores. RNA-seq (a), proteome (b) and metabolome (c) data are shown separately.

Source data are provided as a Source Data file.

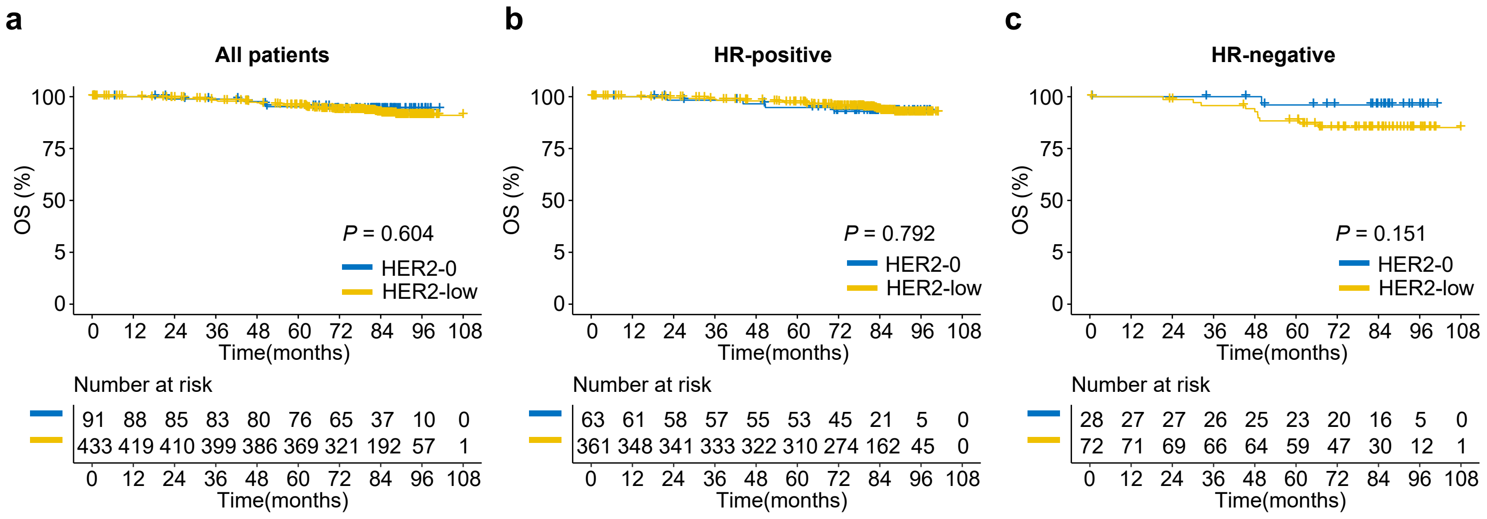
a
All patients
OS (%)
P = 0.604
HER2-0
HER2-low
Time(months)
Number at risk
91 88 85 83 80 76 65 37 10 0
433 419 410 399 386 369 321 192 57 1
b
HR-positive
P = 0.792
63 61 58 57 55 53 45 21 5 0
361 348 341 333 322 310 274 162 45 0
c
HR-negative
P = 0.151
28 27 27 26 25 23 20 16 5 0
72 71 69 66 64 59 47 30 12 1

Figure S4. Difference in overall survival (OS) between HER2-low and HER2-0 patients, related to Figure 2.

(a-c) Kaplan–Meier curves and risk tables showing OS of HER2-low and HER2-0 breast cancers compared by two-sided log-rank test in the entire cohort (a), HR-positive subgroup (b) and HR-negative subgroup (c).

Source data are provided as a Source Data file.

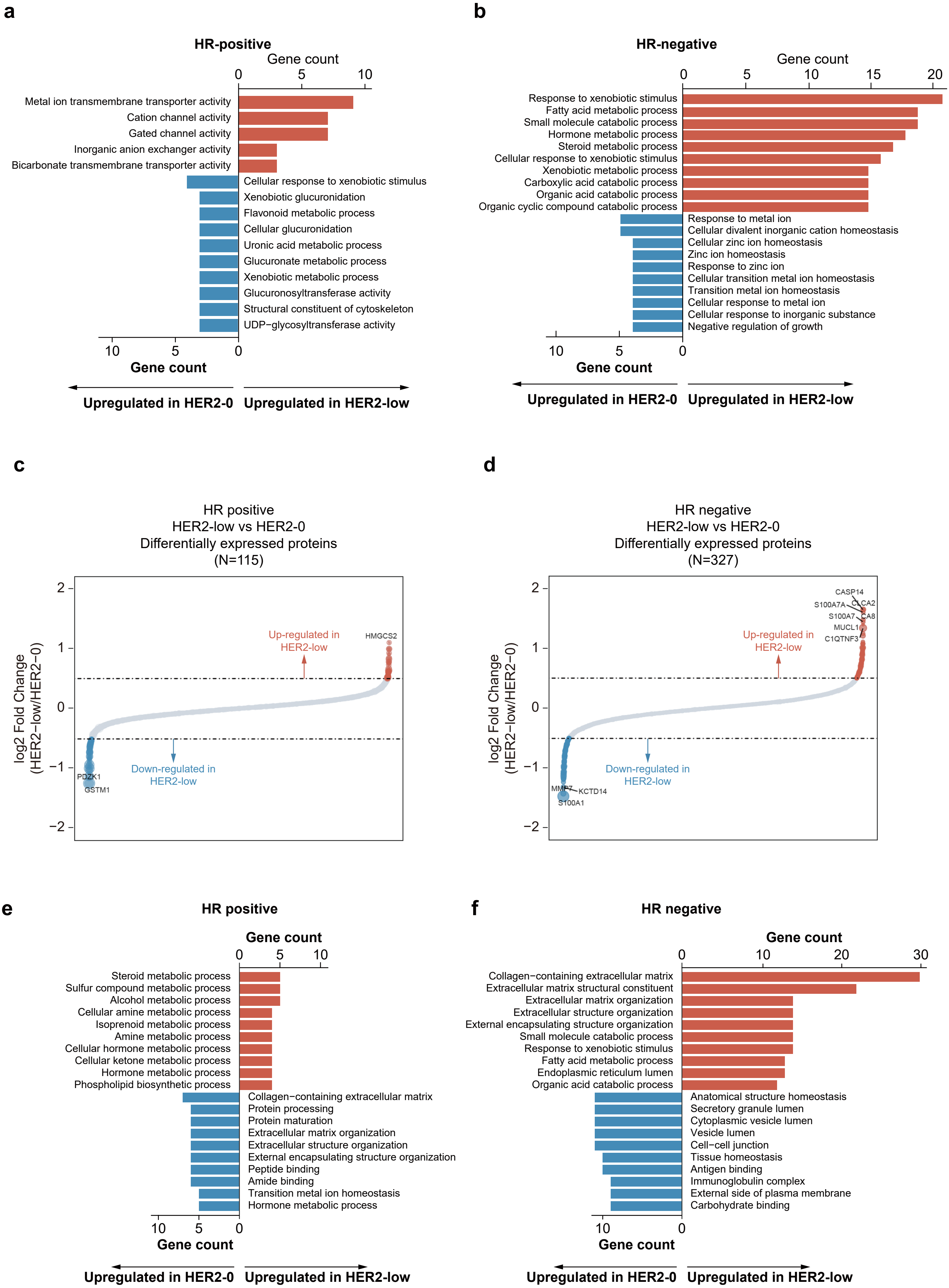
a
HR-positive
Gene count
0 5 10
Metal ion transmembrane transporter activity
Cation channel activity
Gated channel activity
Inorganic anion exchanger activity
Bicarbonate transmembrane transporter activity
Cellular response to xenobiotic stimulus
Xenobiotic glucuronidation
Flavonoid metabolic process
Cellular glucuronidation
Uronic acid metabolic process
Glucuronate metabolic process
Xenobiotic metabolic process
Glucuronosyltransferase activity
Structural constituent of cytoskeleton
UDP−glycosyltransferase activity
10 5 0
Gene count
Upregulated in HER2-0
Upregulated in HER2-low
b
HR-negative
Gene count
0 5 10 15 20
Response to xenobiotic stimulus
Fatty acid metabolic process
Small molecule catabolic process
Hormone metabolic process
Steroid metabolic process
Cellular response to xenobiotic stimulus
Xenobiotic metabolic process
Carboxylic acid catabolic process
Organic acid catabolic process
Organic cyclic compound catabolic process
Response to metal ion
Cellular divalent inorganic cation homeostasis
Cellular zinc ion homeostasis
Zinc ion homeostasis
Response to zinc ion
Cellular transition metal ion homeostasis
Transition metal ion homeostasis
Cellular response to metal ion
Cellular response to inorganic substance
Negative regulation of growth
10 5 0
Gene count
Upregulated in HER2-0
Upregulated in HER2-low
c
HR positive
HER2-low vs HER2-0
Differentially expressed proteins
(N=115)
log2 Fold Change
(HER2−low/HER2−0)
2 1 0 −1 −2
Up-regulated in
HER2-low
HMGCS2
Down-regulated in
HER2-low
PDZK1
GSTM1
d
HR negative
HER2-low vs HER2-0
Differentially expressed proteins
(N=327)
log2 Fold Change
(HER2−low/HER2−0)
2 1 0 −1 −2
CASP14
S100A7A
CLCA2
S100A7
CA8
MUCL1
C1QTNF3
Up-regulated in
HER2-low
Down-regulated in
HER2-low
MMP7
KCTD14
S100A1
e
HR positive
Gene count
0 5 10
Steroid metabolic process
Sulfur compound metabolic process
Alcohol metabolic process
Cellular amine metabolic process
Isoprenoid metabolic process
Amine metabolic process
Cellular hormone metabolic process
Cellular ketone metabolic process
Hormone metabolic process
Phospholipid biosynthetic process
Collagen−containing extracellular matrix
Protein processing
Protein maturation
Extracellular matrix organization
Extracellular structure organization
External encapsulating structure organization
Peptide binding
Amide binding
Transition metal ion homeostasis
Hormone metabolic process
10 5 0
Gene count
Upregulated in HER2-0
Upregulated in HER2-low
f
HR negative
Gene count
0 10 20 30
Collagen−containing extracellular matrix
Extracellular matrix structural constituent
Extracellular matrix organization
Extracellular structure organization
External encapsulating structure organization
Small molecule catabolic process
Response to xenobiotic stimulus
Fatty acid metabolic process
Endoplasmic reticulum lumen
Organic acid catabolic process
Anatomical structure homeostasis
Secretory granule lumen
Cytoplasmic vesicle lumen
Vesicle lumen
Cell−cell junction
Tissue homeostasis
Antigen binding
Immunoglobulin complex
External side of plasma membrane
Carbohydrate binding
10 0
Gene count
Upregulated in HER2-0
Upregulated in HER2-low

Figure S5. Difference between HER2-low and HER2-0 breast cancer with different HR statuses, related to Figure 3.

(a-b) Gene Ontology (GO) enrichment analysis of genes that were differentially expressed between HER2-low and HER2-0 breast cancers in the HR-positive (a) and HR-negative (b) subgroups.

(c-d) Dot plot showing differentially expressed proteins between HER2-low and HER2-0 breast cancers in the HR-positive (c) and HR-negative (d) subgroups.

(e-f) GO enrichment analysis of proteins that were differentially expressed between HER2-low and HER2-0 breast cancers in the HR-positive (e) and HR-negative (f) subgroups.

Source data are provided as a Source Data file.

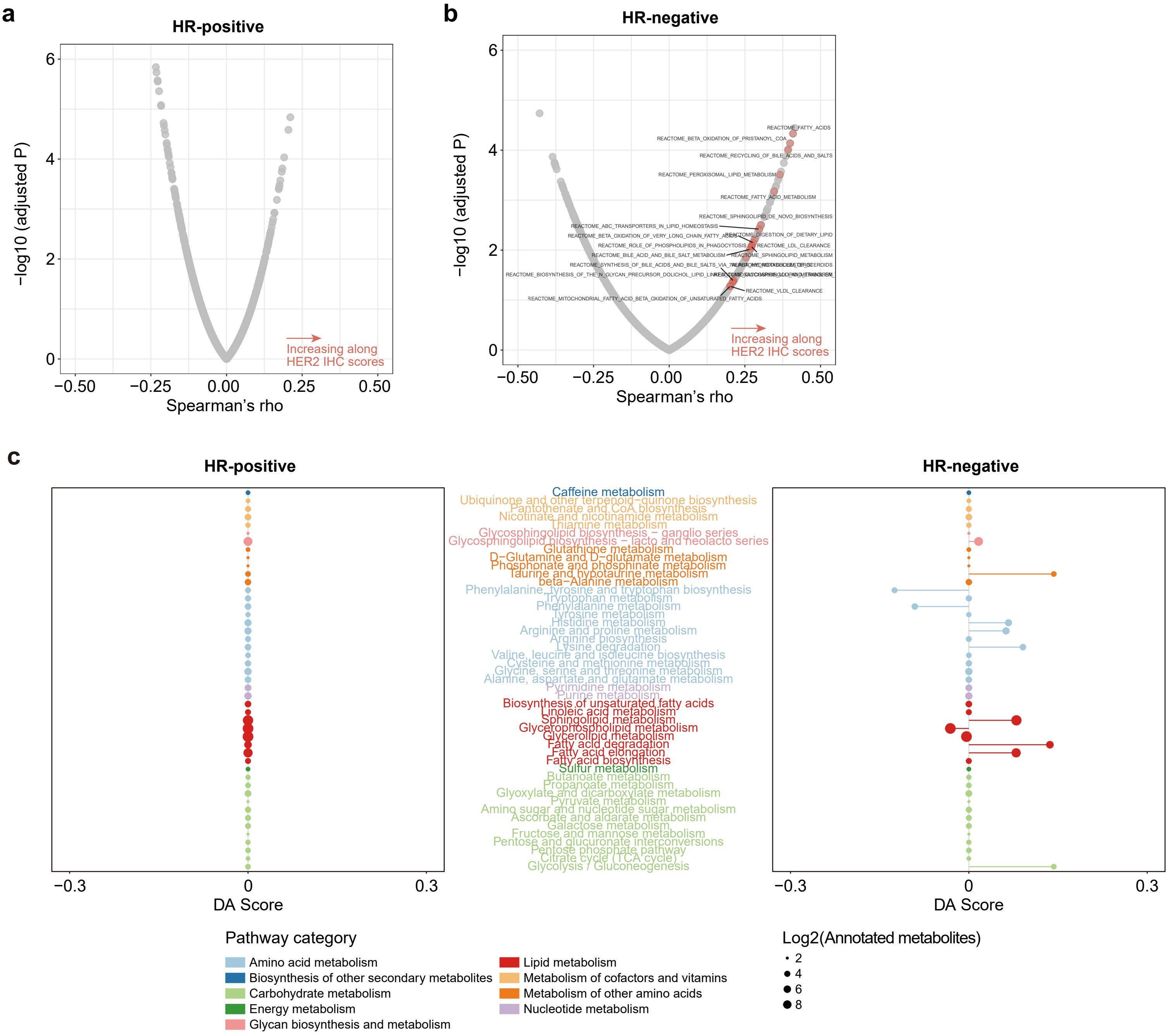

a
HR-positive
−log10 (adjusted P)
0
2
4
6
−0.50
−0.25
0.00
0.25
0.50
Spearman's rho
Increasing along
HER2 IHC scores
b
HR-negative
−log10 (adjusted P)
REACTOME_FATTY_ACIDS
REACTOME_BETA_OXIDATION_OF_PRISTANOYL_COA
REACTOME_RECYCLING_OF_BILE_ACIDS_AND_SALTS
REACTOME_PEROXISOMAL_LIPID_METABOLISM
REACTOME_FATTY_ACID_METABOLISM
REACTOME_SPHINGOLIPID_DE_NOVO_BIOSYNTHESIS
REACTOME_ABC_TRANSPORTERS_IN_LIPID_HOMEOSTASIS
REACTOME_DIGESTION_OF_DIETARY_LIPID
REACTOME_ROLE_OF_PHOSPHOLIPIDS_IN_PHAGOCYTOSIS
REACTOME_LDL_CLEARANCE
REACTOME_BILE_ACID_AND_BILE_SALT_METABOLISM
REACTOME_SPHINGOLIPID_METABOLISM
REACTOME_VLDL_CLEARANCE
REACTOME_MITOCHONDRIAL_FATTY_ACID_BETA_OXIDATION_OF_UNSATURATED_FATTY_ACIDS
Spearman's rho
Increasing along
HER2 IHC scores
c
HR-positive
HR-negative
Caffeine metabolism
Ubiquinone and other terpenoid−quinone biosynthesis
Pantothenate and CoA biosynthesis
Nicotinate and nicotinamide metabolism
Thiamine metabolism
Glycosphingolipid biosynthesis − ganglio series
Glycosphingolipid biosynthesis − lacto and neolacto series
Glutathione metabolism
D−Glutamine and D−glutamate metabolism
Phosphonate and phosphinate metabolism
Taurine and hypotaurine metabolism
beta−Alanine metabolism
Phenylalanine, tyrosine and tryptophan biosynthesis
Tryptophan metabolism
Phenylalanine metabolism
Tyrosine metabolism
Histidine metabolism
Arginine and proline metabolism
Arginine biosynthesis
Lysine degradation
Valine, leucine and isoleucine biosynthesis
Cysteine and methionine metabolism
Glycine, serine and threonine metabolism
Alanine, aspartate and glutamate metabolism
Pyrimidine metabolism
Purine metabolism
Biosynthesis of unsaturated fatty acids
Linoleic acid metabolism
Sphingolipid metabolism
Glycerophospholipid metabolism
Glycerolipid metabolism
Fatty acid degradation
Fatty acid elongation
Fatty acid biosynthesis
Sulfur metabolism
Butanoate metabolism
Propanoate metabolism
Glyoxylate and dicarboxylate metabolism
Pyruvate metabolism
Amino sugar and nucleotide sugar metabolism
Ascorbate and aldarate metabolism
Galactose metabolism
Fructose and mannose metabolism
Pentose and glucuronate interconversions
Pentose phosphate pathway
Citrate cycle (TCA cycle)
Glycolysis / Gluconeogenesis
−0.3
0
0.3
DA Score
Pathway category
Amino acid metabolism
Biosynthesis of other secondary metabolites
Carbohydrate metabolism
Energy metabolism
Glycan biosynthesis and metabolism
Lipid metabolism
Metabolism of cofactors and vitamins
Metabolism of other amino acids
Nucleotide metabolism
Log2(Annotated metabolites)
2
4
6
8

Figure S6. The trend of pathway activity and result of metabolite enrichment, related to Figure 3.

(a-b) Dot plots showing the trend of REACTOME pathway activity changing with HER2 IHC scores in HR-positive (a) and HR-negative (b) subgroups. Significantly increasing or decreasing lipid-related gene sets were plotted in red dots and annotated. P values were computed by Spearman's rank correlation analysis and were adjusted for multiple testing using false discovery rate method.

(c) Pathway-based analysis of lipid and polar metabolite changes along with HER2 IHC scores in different HR subgroups.

Source data are provided as a Source Data file.

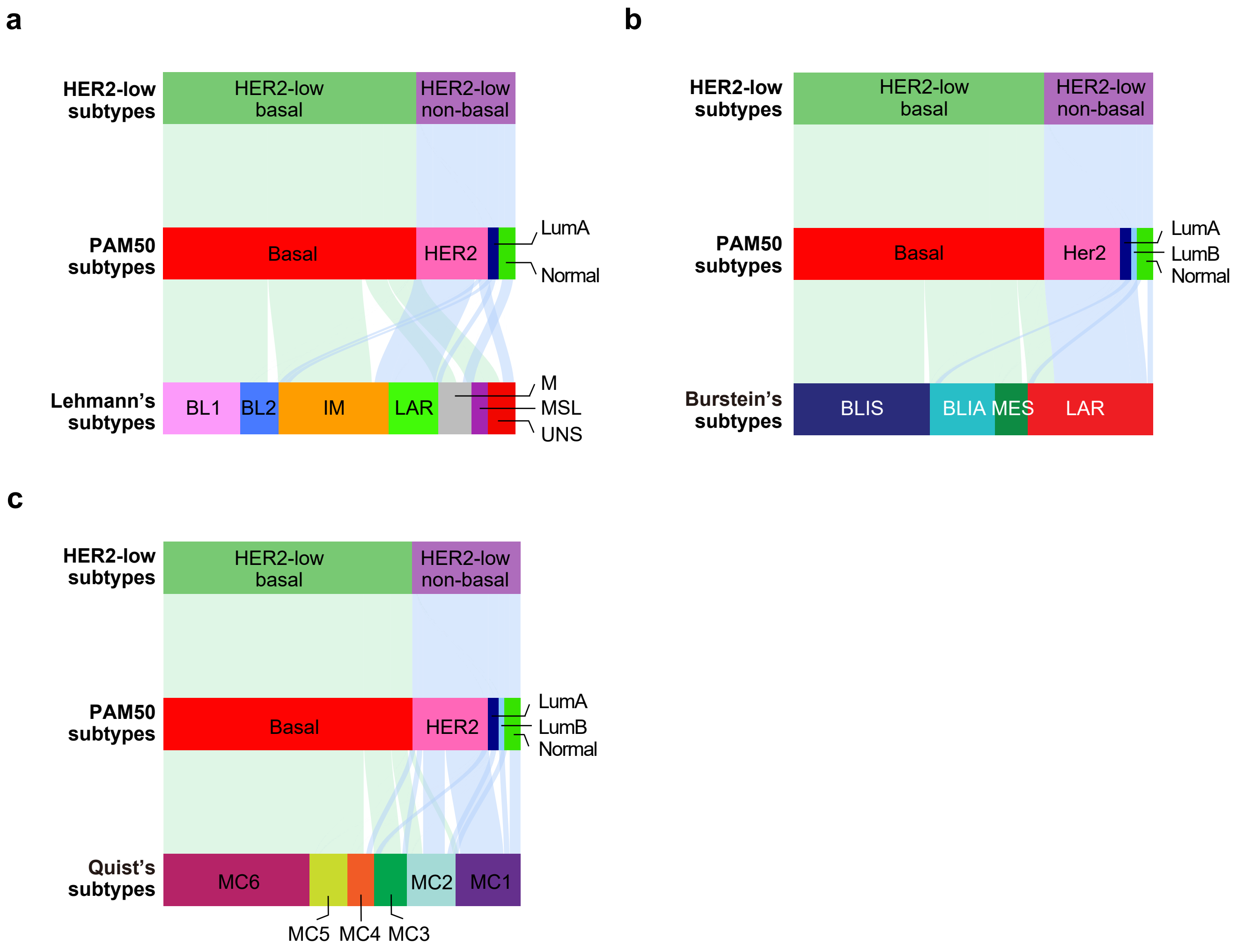
a
HER2-low subtypes
HER2-low basal
HER2-low non-basal
PAM50 subtypes
Basal
HER2
LumA
Normal
Lehmann's subtypes
BL1
BL2
IM
LAR
M
MSL
UNS
b
HER2-low subtypes
HER2-low basal
HER2-low non-basal
PAM50 subtypes
Basal
Her2
LumA
LumB
Normal
Burstein's subtypes
BLIS
BLIA
MES
LAR
c
HER2-low subtypes
HER2-low basal
HER2-low non-basal
PAM50 subtypes
Basal
HER2
LumA
LumB
Normal
Quist's subtypes
MC6
MC2
MC1
MC5
MC4
MC3

Figure S7. Sankey diagram showing the classification of HR-negative HER2-low non-basal-like tumors in the PAM50 subtype and other molecular subtypes, related to Figure 4.

(a) Lehmann's TNBC subtyping[1]. (b) Burstein's TNBC subtyping[2]. (c) Quist's TNBC subtyping[3]

Source data are provided as a Source Data file.

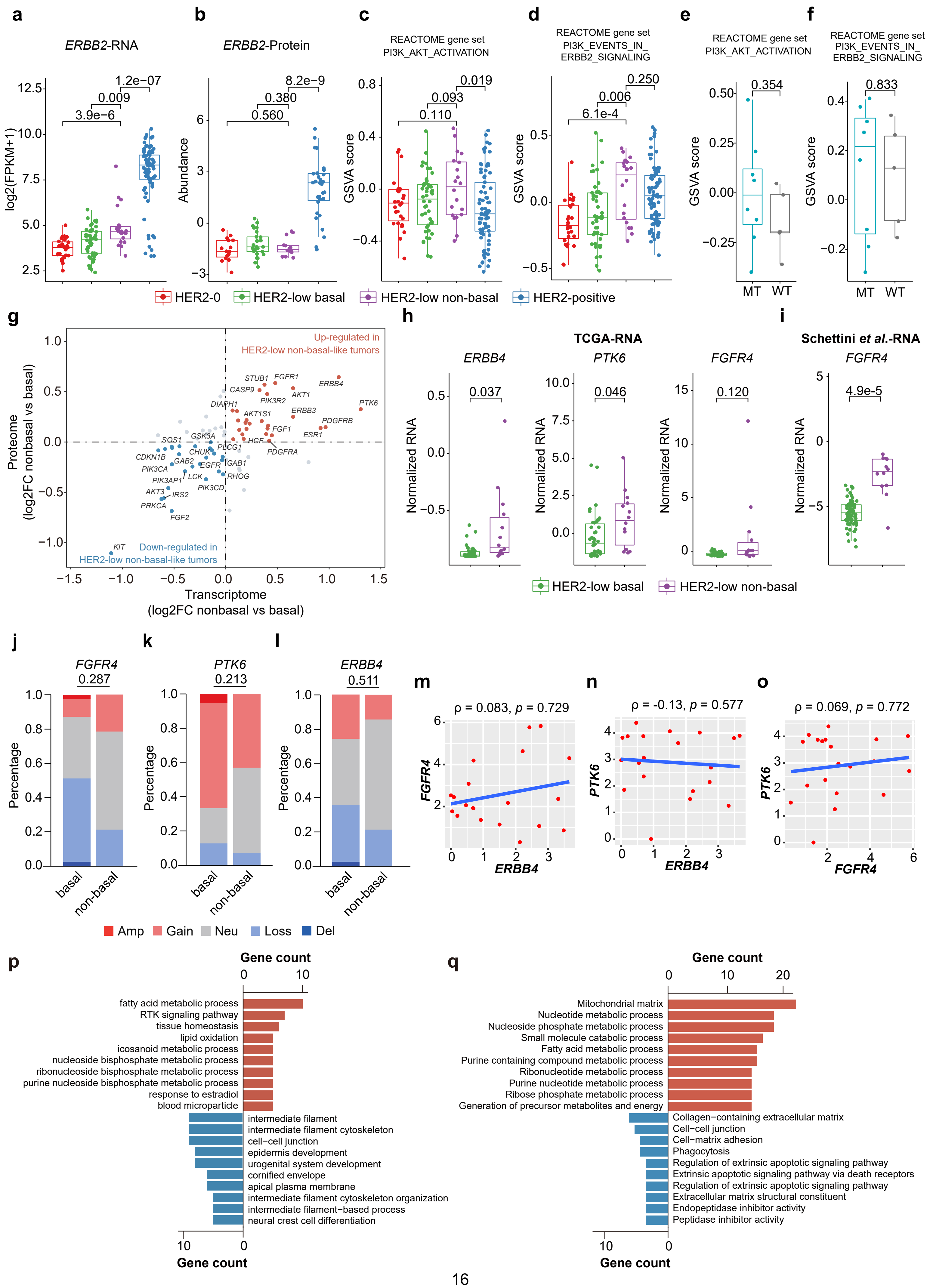
a
ERBB2-RNA
log2(FPKM+1)
3.9e−6
0.009
1.2e−07
b
ERBB2-Protein
Abundance
0.560
0.380
8.2e−9
c
REACTOME gene set
PI3K_AKT_ACTIVATION
GSVA score
0.110
0.093
0.019
d
REACTOME gene set
PI3K_EVENTS_IN_
ERBB2_SIGNALING
GSVA score
6.1e−4
0.006
0.250
e
REACTOME gene set
PI3K_AKT_ACTIVATION
GSVA score
0.354
MT
WT
f
REACTOME gene set
PI3K_EVENTS_IN_
ERBB2_SIGNALING
GSVA score
0.833
MT
WT
HER2-0
HER2-low basal
HER2-low non-basal
HER2-positive
g
Up-regulated in
HER2-low non-basal-like tumors
Down-regulated in
HER2-low non-basal-like tumors
Proteome
(log2FC nonbasal vs basal)
Transcriptome
(log2FC nonbasal vs basal)
STUB1
FGFR1
ERBB4
CASP9
AKT1
PTK6
DIAPH1
PIK3R2
ERBB3
AKT1S1
PDGFRB
FGF1
ESR1
SOS1
GSK3A
HGF
CHUK
PLCG1
PDGFRA
CDKN1B
GAB2
EGFR
IGAB1
PIK3CA
LCK
RHOG
PIK3AP1
PIK3CD
AKT3
IRS2
PRKCA
FGF2
KIT
h
TCGA-RNA
ERBB4
0.037
PTK6
0.046
FGFR4
0.120
Normalized RNA
i
Schettini et al.-RNA
FGFR4
4.9e-5
Normalized RNA
HER2-low basal
HER2-low non-basal
j
FGFR4
0.287
k
PTK6
0.213
l
ERBB4
0.511
Percentage
basal
non-basal
Amp
Gain
Neu
Loss
Del
m
ρ = 0.083, p = 0.729
FGFR4
ERBB4
n
ρ = -0.13, p = 0.577
PTK6
ERBB4
o
ρ = 0.069, p = 0.772
PTK6
FGFR4
p
Gene count
fatty acid metabolic process
RTK signaling pathway
tissue homeostasis
lipid oxidation
icosanoid metabolic process
nucleoside bisphosphate metabolic process
ribonucleoside bisphosphate metabolic process
purine nucleoside bisphosphate metabolic process
response to estradiol
blood microparticle
intermediate filament
intermediate filament cytoskeleton
cell−cell junction
epidermis development
urogenital system development
cornified envelope
apical plasma membrane
intermediate filament cytoskeleton organization
intermediate filament−based process
neural crest cell differentiation
Gene count
q
Gene count
Mitochondrial matrix
Nucleotide metabolic process
Nucleoside phosphate metabolic process
Small molecule catabolic process
Fatty acid metabolic process
Purine containing compound metabolic process
Ribonucleotide metabolic process
Purine nucleotide metabolic process
Ribose phosphate metabolic process
Generation of precursor metabolites and energy
Collagen−containing extracellular matrix
Cell−cell junction
Cell−matrix adhesion
Phagocytosis
Regulation of extrinsic apoptotic signaling pathway
Extrinsic apoptotic signaling pathway via death receptors
Regulation of extrinsic apoptotic signaling pathway
Extracellular matrix structural constituent
Endopeptidase inhibitor activity
Peptidase inhibitor activity
Gene count

Figure S8. Internal molecular heterogeneity of HR-negative HER2-low breast cancers, related to Figure 4.

(a-b) Boxplot comparing the mRNA (a) and protein (b) levels of *ERBB2* among HER2 subgroups. For RNA, the number (N) of HER2-0, HER2-low basal-like, HER2-low non-basal-like, and HER2-positive is 27, 46, 20 and 81. For protein, the number (N) was 15, 26, 14 and 31. P values were computed using the two-sided Wilcoxon test.

(c-d) Boxplots comparing the enrichment score of *REACTOME PI3K AKT ACTIVATION* (c) or *PI3K EVENTS IN ERBB2 SIGNALING* (d) among HER2 subgroups using gene set variation analysis (GSVA). The number (N) of HER2-0, HER2-low basal-like, HER2-low non-basal-like, and HER2-positive is 27, 46, 20 and 81. P values were computed using the two-sided Wilcoxon test.

(e-f) Boxplot comparing the enrichment score of *REACTOME PI3K AKT ACTIVATION* (e) and *PI3K EVENTS IN ERBB2 SIGNALING* (f) between *PIK3CA* wild-type (WT, N=5) and mutated samples (MT, N=8) among tumor subgroups. P values were computed using the two-sided Wilcoxon test.

(g) Dot plot showing the log2(fold change) of the comparison of genes involved in PI3K and *ERBB2* signaling between HR-negative HER2-low non-basal-like tumors and basal-like tumors at both the RNA and protein levels.

(h) Expression of *ERBB4, PTK6* and *FGFR4* among HER2 subgroups in the TCGA-BRCA cohort[4,5]. The number (N) of HER2-low basal-like and HER2-low non-basal-like is 42 and 14. P values were computed using the two-sided Wilcoxon test.

(i) Expression of *FGFR4* among HER2 subgroups in Schettini *et al.*'s cohort[6]. The number (N) of HER2-low basal-like and HER2-low non-basal-like is 65 and 12. P values were computed using the two-sided Wilcoxon test.

(j-l) Bar plots comparing the copy number alterations (CNAs) of *FGFR4* (j), *PTK6* (k) and *ERBB4* (l) between non-basal-like tumors and basal-like tumors. P values were computed using the two-sided Fisher's exact test.

(m-o) Dot plot of the Spearman correlation between the expression of *ERBB4* and *FGFR4* (m), *ERBB4* and *PTK6* (n), and *PTK6* and *FGFR4* (o). P values were computed by Spearman correlation analysis.

(p-q) Gene Ontology (GO) enrichment analysis of genes (p) and proteins (q) that were differentially expressed between HER2-low non-basal-like and basal-like tumors.

Basal: basal-like; non-basal: non-basal-like.

In boxplots, the centerline represents the median, the box limits represent the upper and lower quartiles, the whiskers represent the 1.5x interquartile range, and the points represent individual samples.

Source data are provided as a Source Data file.

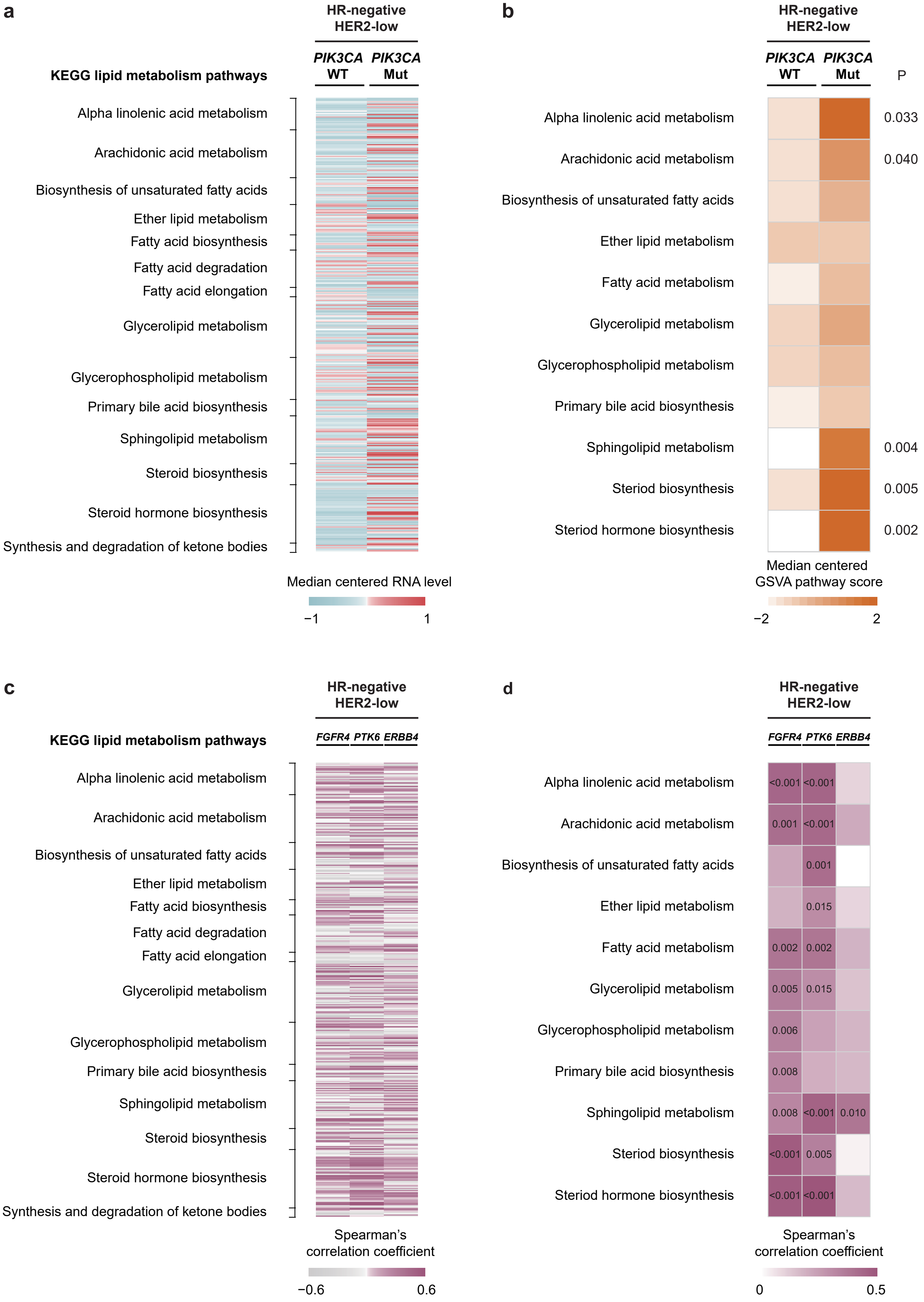
a
HR-negative HER2-low
PIK3CA WT
PIK3CA Mut
KEGG lipid metabolism pathways
Alpha linolenic acid metabolism
Arachidonic acid metabolism
Biosynthesis of unsaturated fatty acids
Ether lipid metabolism
Fatty acid biosynthesis
Fatty acid degradation
Fatty acid elongation
Glycerolipid metabolism
Glycerophospholipid metabolism
Primary bile acid biosynthesis
Sphingolipid metabolism
Steroid biosynthesis
Steroid hormone biosynthesis
Synthesis and degradation of ketone bodies
Median centered RNA level
−1
1
b
HR-negative HER2-low
PIK3CA WT
PIK3CA Mut
P
Alpha linolenic acid metabolism 0.033
Arachidonic acid metabolism 0.040
Biosynthesis of unsaturated fatty acids
Ether lipid metabolism
Fatty acid metabolism
Glycerolipid metabolism
Glycerophospholipid metabolism
Primary bile acid biosynthesis
Sphingolipid metabolism 0.004
Steriod biosynthesis 0.005
Steriod hormone biosynthesis 0.002
Median centered GSVA pathway score
−2
2
c
HR-negative HER2-low
FGFR4
PTK6
ERBB4
KEGG lipid metabolism pathways
Alpha linolenic acid metabolism
Arachidonic acid metabolism
Biosynthesis of unsaturated fatty acids
Ether lipid metabolism
Fatty acid biosynthesis
Fatty acid degradation
Fatty acid elongation
Glycerolipid metabolism
Glycerophospholipid metabolism
Primary bile acid biosynthesis
Sphingolipid metabolism
Steroid biosynthesis
Steroid hormone biosynthesis
Synthesis and degradation of ketone bodies
Spearman's correlation coefficient
−0.6
0.6
d
HR-negative HER2-low
FGFR4
PTK6
ERBB4
Alpha linolenic acid metabolism <0.001 <0.001
Arachidonic acid metabolism 0.001 <0.001
Biosynthesis of unsaturated fatty acids 0.001
Ether lipid metabolism 0.015
Fatty acid metabolism 0.002 0.002
Glycerolipid metabolism 0.005 0.015
Glycerophospholipid metabolism 0.006
Primary bile acid biosynthesis 0.008
Sphingolipid metabolism 0.008 <0.001 0.010
Steriod biosynthesis <0.001 0.005
Steriod hormone biosynthesis <0.001 <0.001
Spearman's correlation coefficient
0
0.5

Figure S9. The association between molecular characteristics and lipid metabolism in HR-negative HER2-low breast cancers, related to Figure 4.

(a) Heatmap comparing the gene expression level of lipid metabolism between *PIK3CA*-mutated and *PIK3CA*-wild-type breast cancers.

(b) Heatmap comparing the pathway score of lipid metabolism between *PIK3CA*-mutated and *PIK3CA*-wild-type breast cancers. Original p values derived from two-sided Wilcoxon test that is less than 0.05 were annotated.

(c) Heatmap showing Spearman's correlation coefficient between the expression levels of *FGFR4/PTK6/ERBB4* and lipid metabolism-related genes.

(d) Heatmap showing Spearman's correlation coefficient between the expression levels of *FGFR4/PTK6/ERBB4* and the pathway scores of lipid metabolism. Pathways that were significantly correlated with *FGFR4/PTK6/ERBB4* levels (FDR from two-sided Spearman's correlation coefficient were less than 0.05) are marked with the exact FDR.

Source data are provided as a Source Data file.

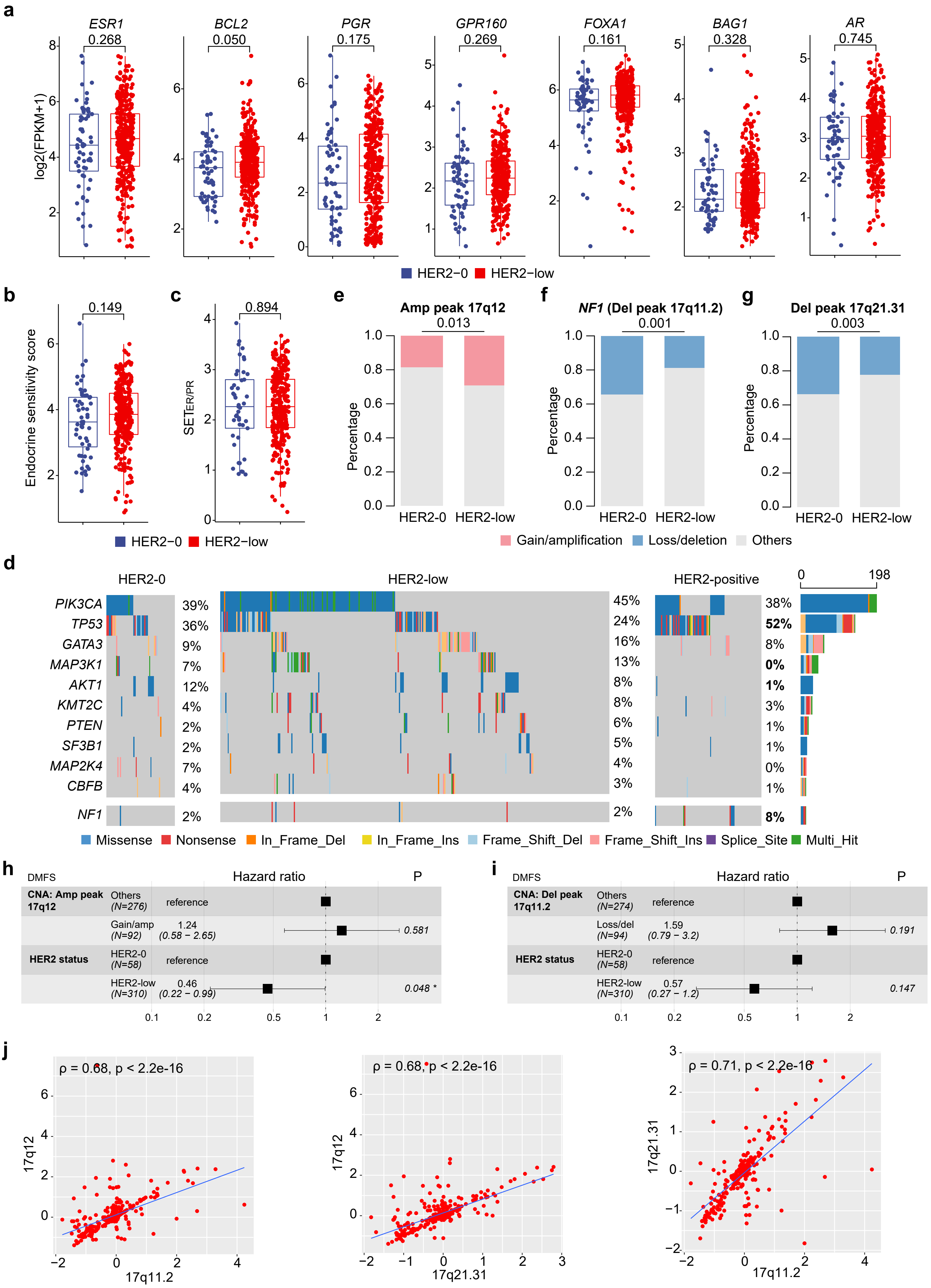

a
ESR1 0.268
BCL2 0.050
PGR 0.175
GPR160 0.269
FOXA1 0.161
BAG1 0.328
AR 0.745
log2(FPKM+1)
HER2−0 HER2−low
b
0.149
Endocrine sensitivity score
c
0.894
SETER/PR
HER2−0 HER2−low
e
Amp peak 17q12
0.013
f
NF1 (Del peak 17q11.2)
0.001
g
Del peak 17q21.31
0.003
Percentage
HER2-0 HER2-low
Gain/amplification Loss/deletion Others
d
HER2-0
HER2-low
HER2-positive
PIK3CA 39% 45% 38%
TP53 36% 24% 52%
GATA3 9% 16% 8%
MAP3K1 7% 13% 0%
AKT1 12% 8% 1%
KMT2C 4% 8% 3%
PTEN 2% 6% 1%
SF3B1 2% 5% 1%
MAP2K4 7% 4% 0%
CBFB 4% 3% 1%
NF1 2% 2% 8%
0 198
Missense Nonsense In_Frame_Del In_Frame_Ins Frame_Shift_Del Frame_Shift_Ins Splice_Site Multi_Hit
h
DMFS
Hazard ratio
P
CNA: Amp peak 17q12
Others (N=276) reference
Gain/amp (N=92) 1.24 (0.58 − 2.65) 0.581
HER2 status
HER2-0 (N=58) reference
HER2-low (N=310) 0.46 (0.22 − 0.99) 0.048 *
0.1 0.2 0.5 1 2
i
DMFS
Hazard ratio
P
CNA: Del peak 17q11.2
Others (N=274) reference
Loss/del (N=94) 1.59 (0.79 − 3.2) 0.191
HER2 status
HER2-0 (N=58) reference
HER2-low (N=310) 0.57 (0.27 − 1.2) 0.147
0.1 0.2 0.5 1 2
j
ρ = 0.68, p < 2.2e-16
17q12
17q11.2
ρ = 0.68, p < 2.2e-16
17q12
17q21.31
ρ = 0.71, p < 2.2e-16
17q21.31
17q11.2

Figure S10. Molecular difference between HER2-low and HER2-0 tumors in the HR-positive subgroup and its relation with survival, related to Figure 5.

(a-c) Boxplots comparing luminal-related genes[6] (b), endocrine sensitivity score[7] (c) and $SET_{ER/PR}$ score[8] (d). In boxplots, the centerline represents the median, the box limits represent the upper and lower quartiles, the whiskers represent the 1.5x interquartile range, and the points represent individual samples. For luminal-related genes, the number (N) of HER2-0 and HER2-low is 61 and 355. For endocrine sensitivity score, the number (N) was 55 and 301. For $SET_{ER/PR}$ score, the number (N) was 57 and 282. P values were computed using the two-sided Wilcoxon test.

(d) Somatic mutations of cancer-related genes (CAGs) among HER2 status subgroups. Upper panel: top 10 frequently mutated CAGs, lower panel: other differentially mutated CAGs between HER2 status subgroups. Genes that were differentially mutated (P<0.05) between HER2-positive and HER2-0 tumors compared with HER2-low tumors are in bold font. P values were computed using the two-sided Fisher's exact test.

(e-g) Comparison of the copy number of Amp peak 17q12 (e), Del peak 17q11.12 (f) and Del peak 17q21.31 (g). P values were computed using the two-sided Fisher's exact test.

(h-i) Forest plots showing the multivariable Cox regression analysis for distant metastasis-free survival (DMFS) of the status HR and the status of focal peaks 17q12 (h) and 17q11.2 (i) in HR-positive HER2-low breast cancers. Number(N) of patients belonging to each category is indicated. Association of all variables with prognosis is analyzed using a two-sided Cox proportional hazard regression analysis. Error bars represent the 95% confidence interval of hazard ratio.

(j) Spearman's correlation between the copy numbers of 17q11.2, 17q21.31 and 17q12. P values were computed by Spearman's rank correlation analysis.

Gain/amp: gain/amplification; Loss/del: loss/deletion.

Source data are provided as a Source Data file.

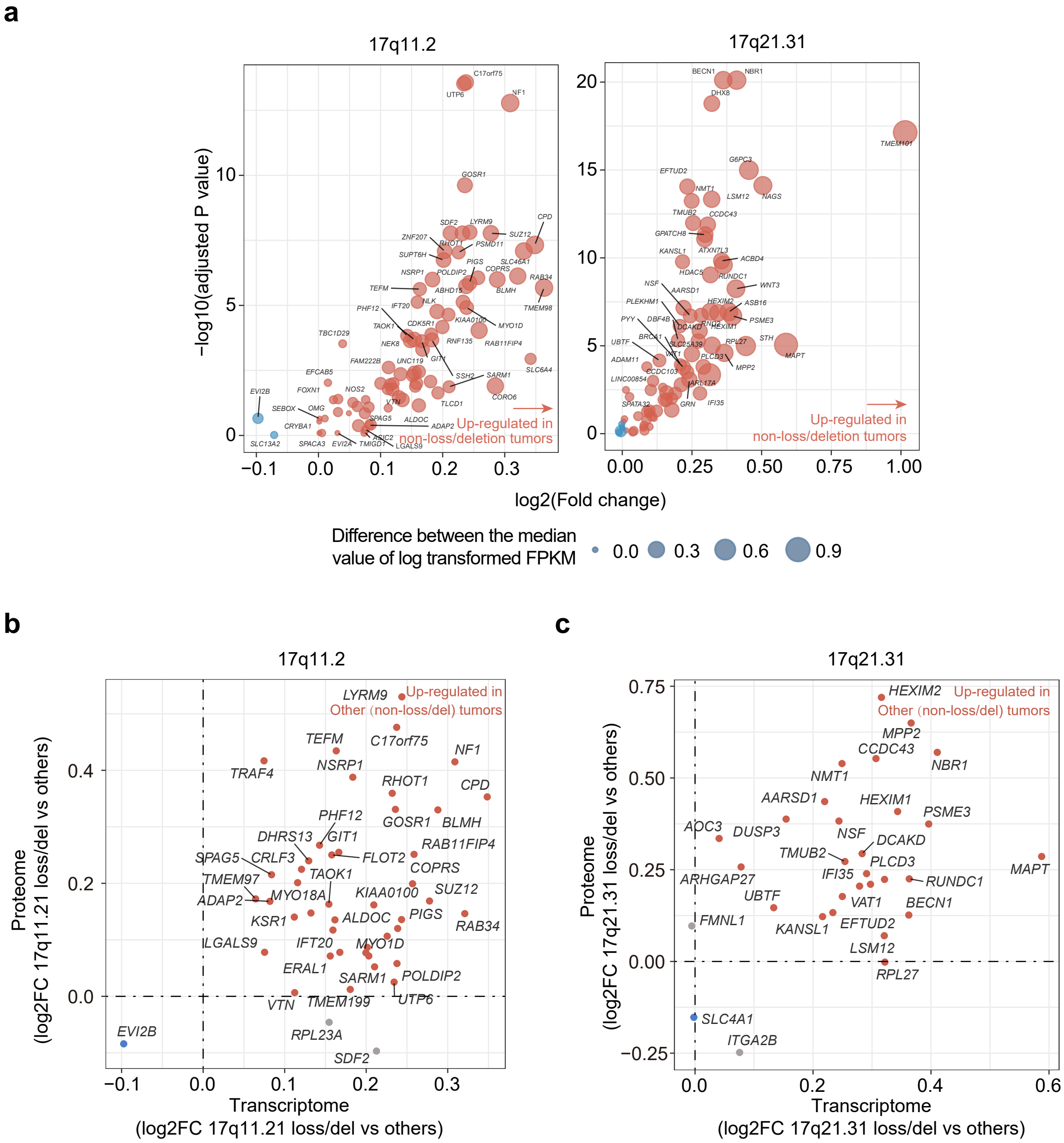
a
17q11.2
17q21.31
−log10(adjusted P value)
log2(Fold change)
Up-regulated in non-loss/deletion tumors
Difference between the median value of log transformed FPKM
0.0 0.3 0.6 0.9
b
17q11.2
Up-regulated in Other (non-loss/del) tumors
Proteome (log2FC 17q11.21 loss/del vs others)
Transcriptome (log2FC 17q11.21 loss/del vs others)
LYRM9
TEFM
C17orf75
NF1
TRAF4
NSRP1
RHOT1
CPD
PHF12
GOSR1
BLMH
DHRS13
GIT1
RAB11FIP4
SPAG5
CRLF3
FLOT2
COPRS
TMEM97
TAOK1
ADAP2
MYO18A
KIAA0100
SUZ12
KSR1
ALDOC
PIGS
RAB34
LGALS9
IFT20
MYO1D
ERAL1
SARM1
POLDIP2
VTN
TMEM199
UTP6
EVI2B
RPL23A
SDF2
c
17q21.31
Up-regulated in Other (non-loss/del) tumors
Proteome (log2FC 17q21.31 loss/del vs others)
Transcriptome (log2FC 17q21.31 loss/del vs others)
HEXIM2
MPP2
CCDC43
NBR1
NMT1
AARSD1
HEXIM1
PSME3
AOC3
DUSP3
NSF
DCAKD
TMUB2
PLCD3
MAPT
ARHGAP27
IFI35
RUNDC1
UBTF
VAT1
BECN1
FMNL1
KANSL1
EFTUD2
LSM12
RPL27
SLC4A1
ITGA2B

Figure S11. Downstream effect of loss/deletion in 17q11.2 and 17q21.31 within the HR-positive HER2-negative subgroup, related to Figure 5.

(a) Comparison of the expression level of genes located in 17q11.2 and 17q21.31 between loss/deletion patients and others. P values were computed using the two-sided Wilcoxon test and were adjusted for multiple testing using false discovery rate method.

(b-c) Dot plot showing the log2(fold change) of the comparison of genes located in 17q11.2 (b) and 17q21.31 (c) between tumors with or without loss/deletion in corresponding peaks at both the RNA and protein levels.

Source data are provided as a Source Data file.

Table S1. Baseline characteristics of included patients stratified by HER2 status.

		Overall N=707	HER2-0 N=91	HER2-low N=434	HER2-positive N=182	*P1*	*P2*	*P3*
Clinicopathological characteristics								
Age (years)								
	Mean (SD)	53.00 (10.40)	53.57 (11.95)	53.26 (10.49)	52.07 (9.30)	0.364	0.805	0.182
Age (years, %)								
	<40	61 (8.6)	12 (13.2)	33 (7.6)	16 (8.8)	0.109	0.184	0.143
	40-59	466 (65.9)	53 (58.2)	283 (65.2)	130 (71.4)			
	≥60	180 (25.5)	26 (28.6)	118 (27.2)	36 (19.8)			
Menopause (%)								
	Male	1 (0.1)	0 (0.0)	1 (0.2)	0 (0.0)	0.810	0.416	0.670
	No	289 (40.9)	33 (36.3)	184 (42.5)	72 (39.6)			
	Yes	416 (58.9)	58 (63.7)	248 (57.3)	110 (60.4)			
	NA	1	0	1	0			
Laterality (%)								
	Left	370 (52.3)	48 (52.7)	217 (50.0)	105 (57.7)	0.216	0.646	0.093
	Right	337 (47.7)	43 (47.3)	217 (50.0)	77 (42.3)			
HER2-low status full (%)								
	HER2 0	91 (12.9)	91 (100.0)	0 (0.0)	0 (0.0)	/	/	/
	HER2 1+	227 (32.1)	0 (0.0)	227 (52.3)	0 (0.0)			
	HER2 2+ ISH-	207 (29.3)	0 (0.0)	207 (47.7)	0 (0.0)			
	HER2 positive	182 (25.7)	0 (0.0)	0 (0.0)	182 (100.0)			
Histology (%)								
	IDC	675 (95.5)	88 (96.7)	408 (94.0)	179 (98.4)	0.048	0.121	0.090
	ILC	18 (2.5)	0 (0.0)	16 (3.7)	2 (1.1)			
	Other	14 (2.0)	3 (3.3)	10 (2.3)	1 (0.5)			
Grade (%)								
	<3	350 (52.2)	43 (48.9)	250 (61.7)	57 (32.0)	1.9e-10	0.031	3.4e-11
	3	321 (47.8)	45 (51.1)	155 (38.3)	121 (68.0)			
	NA	36	3	29	4			
Ki67 percentage (%)								
	<20	142 (20.1)	20 (22.0)	112 (25.8)	10 (5.5)	2.4e-9	0.507	4.8e-10
	≥20	565 (79.9)	71 (78.0)	322 (74.2)	172 (94.5)			
HR status (%)								
	Positive	525 (74.3)	63 (69.2)	361 (83.2)	101 (55.5)	7.8e-12	0.003	2.8e-12
	Negative	182 (25.7)	28 (30.8)	73 (16.8)	81 (44.5)			
sTILs								
	Mean (SD)	0.17 (0.15)	0.19 (0.16)	0.15 (0.14)	0.21 (0.15)	4.7e-5	0.052	9.6e-6
iTILs								
	Mean (SD)	0.02 (0.02)	0.03 (0.03)	0.02 (0.02)	0.02 (0.03)	0.056	0.031	0.504
pT (%)								
	pT1	310 (44.0)	37 (40.7)	201 (46.5)	72 (39.6)	0.627	0.563	0.246
	pT2	383 (54.3)	53 (58.2)	224 (51.9)	106 (58.2)			
	pT3	12 (1.7)	1 (1.1)	7 (1.6)	4 (2.2)			
	NA	2	0	2	0			
pN (%)								
	pN0	353 (51.6)	52 (61.2)	212 (50.8)	89 (48.9)	0.081	0.343	0.090
	pN1	188 (27.5)	21 (24.7)	120 (28.8)	47 (25.8)			
	pN2	86 (12.6)	7 (8.2)	57 (13.7)	22 (12.1)			
	pN3	57 (8.3)	5 (5.9)	28 (6.7)	24 (13.2)			
	NA	23	6	17	0			
Molecular features								
TMB (mutations/Mb)								
	Mean (SD)	1.45 (1.89)	1.47 (2.27)	1.33 (1.79)	1.71 (1.90)	0.114	0.553	0.032
HRD score								
	Mean (SD)	24.36 (16.57)	28.54 (18.51)	22.81 (16.86)	26.41 (13.30)	0.015	0.014	0.057
PAM50 (%)								
	LumA	202 (29.3)	25 (28.4)	159 (37.8)	18 (9.9)	<2.2e-16	3.5e-4	<2.2e-16
	LumB	200 (29.0)	24 (27.3)	141 (33.5)	35 (19.3)			
	HER2	140 (20.3)	4 (4.5)	36 (8.6)	100 (55.2)			
	Basal	105 (15.2)	31 (35.2)	58 (13.8)	16 (8.8)			
	Normal	43 (6.2)	4 (4.5)	27 (6.4)	12 (6.6)			
	NA	17	3	13	1			

Not available (NA) values in categorical variables were shown but not included in the statistical analysis. Statistical tests of continuous variables were performed using the two-sided Kruskal–Wallis rank sum test. Statistical tests of categorical variables were performed using the two-sided Fisher's exact test. *P1*: comparison among HER2-0, HER2-low and HER2-positive. *P2*: comparison between HER2-0 and HER2-low. *P3*: comparison between HER2-low and HER2-positive. IDC: invasive ductal carcinoma; ILC: invasive lobular carcinoma; ISH: *in situ* hybridization; HR: hormone receptor; sTILs: stromal tumor infiltrating lymphocytes; iTILs: intratumoral tumor infiltrating lymphocytes; TMB: tumor mutation burden; HRD: homologous recombination deficiency; LumA: luminal A; LumB: luminal B; HER2 (in PAM50 section): HER2-enriched; Basal: basal-like; Normal: normal-like; SD: standard deviation.

Table S2. Molecular features and treatment information of HER2-low and HER2-0 breast cancers stratified by hormone receptor (HR) status

		HR-positive			HR-negative		
		HER2-0<br>N=63	HER2-low<br>N=361	*P*	HER2-0<br>N=28	HER2-low<br>N=73	*P*
Molecular features							
TMB (mutations/Mb)							
	Mean (SD)	0.93 (0.92)	1.16 (1.73)	0.327	2.86 (3.73)	2.33 (1.85)	0.398
HRD score							
	Mean (SD)	20.47 (15.39)	19.31 (14.33)	0.623	45.05 (12.48)	40.98 (17.47)	0.329
PAM50 (%)							
	LumA	25 (41.0)	157 (44.2)	0.534	0 (0.0)	2 (3.0)	0.015
	LumB	23 (37.7)	140 (39.4)		1 (3.7)	1 (1.5)	
	HER2	4 (6.6)	22 (6.2)		0 (0.0)	14 (21.2)	
	Basal	5 (8.2)	12 (3.4)		26 (96.3)	46 (69.7)	
	Normal	4 (6.6)	24 (6.8)		0 (0.0)	3 (4.5)	
	NA	2	6		1	7	
Treatment information							
Adjuvant chemotherapy (%)							
	No	9 (14.8)	76 (22.6)	0.234	1 (3.6)	2 (2.9)	1
	Yes	52 (85.2)	261 (77.4)		27 (96.4)	66 (97.1)	
	NA	2	24		0	5	
Taxane usage (%)							
	No	15 (30.0)	108 (34.0)	0.632	3 (15.8)	16 (23.2)	0.753
	Yes	35 (70.0)	210 (66.0)		16 (84.2)	53 (76.8)	
	NA	13	43		9	4	
Anthracycline usage (%)							
	No	24 (48.0)	144 (45.3)	0.761	7 (36.8)	15 (21.7)	0.232
	Yes	26 (52.0)	174 (54.7)		12 (63.2)	54 (78.3)	
	NA	13	43		9	4	
Platinum usage (%)							
	No	50 (100.0)	318 (100.0)	/	16 (84.2)	63 (91.3)	0.399
	Yes	0 (0.0)	0 (0.0)		3 (15.8)	6 (8.7)	
	NA	13	43		9	4	
Capecitabine usage (%)							
	No	49 (98.0)	316 (99.4)	0.356	17 (89.5)	63 (91.3)	1
	Yes	1 (2.0)	2 (0.6)		2 (10.5)	6 (8.7)	
	NA	13	43		9	4	
Adjuvant radiotherapy (%)							
	No	42 (67.7)	208 (65.4)	0.771	22 (78.6)	56 (77.8)	1
	Yes	20 (32.3)	110 (34.6)		6 (21.4)	16 (22.2)	
	NA	1	43		0	1	
Adjuvant endocrine therapy (%)							
	No	2 (3.4)	5 (1.6)	0.311	28 (100.0)	72 (98.6)	1
	Yes	57 (96.6)	305 (98.4)		0 (0.0)	1 (1.4)	
	NA	4	51		0	0	

Not available (NA) values in categorical variables were shown but not included in the statistical analysis. Statistical tests of continuous variables were performed using the Kruskal–Wallis rank sum test. Statistical tests of categorical variables were performed using Fisher's exact test or the chi-square test, where appropriate.HR: hormone receptor; TMB: tumor mutation burden; HRD: homologous recombination deficiency; LumA: luminal A; LumB: luminal B; HER2 (in PAM50 section): HER2-enriched; basal: basal-like; normal: normal-like; SD: standard deviation.

Supplementary Methods

Sample processing for genomic DNA and total RNA extraction

Fresh frozen tumor tissues were macrodissected to prevent the influence of stromal tissues (<30% stromal tissue). The percentage of tumor cells was confirmed to be 50% or more in all breast cancer specimens. We purified genomic DNA from fresh frozen samples and peripheral blood cells using TGuide M24 (Tiangen, Beijing, China). The purity and quantity of genomic DNA were estimated by measuring the absorbance at 260 nm (A260) and 280 nm (A280) using a NanoDrop 2000 spectrophotometer (Thermo Scientific, Wilmington, DE, USA). The extracted DNA was considered pure and suitable for future experiments when the A260/A280 ratio was within the range of 1.6-1.9. We purified the total RNA from tissues that had been previously stored in RNAlater solution using the miRNeasy Mini Kit (Qiagen #217004) according to the manufacturer's instructions. RNA integrity was assessed by an Agilent 4200 Bioanalyzer using RNA ScreenTape (Agilent Inc.), and concentrations were determined by a NanoDrop ND-8000 spectrophotometer (Thermo Fisher Scientific Inc.).

RNA sequencing

Sample preparation and data generation

Libraries were constructed by ribosomal RNA depletion methods. Ribosomal RNA was depleted using a Ribo-off rRNA Depletion Kit (H/M/R) (Vazyme #N406, Vazyme Biotech Co., Ltd., Nanjing, China), and RNA libraries were constructed using a VAHTS Universal V8 RNA-seq Library Prep Kit for Illumina (Vazyme #NR605, Vazyme Biotech Co., Ltd., Nanjing, China). Specifically, the fragmented RNAs were reverse-transcribed into cDNA, and then 3-terminal poly(A) modification was completed. Subsequently, adapters were added to the cDNA. PCR was then used to amplify the libraries. During quality control (QC) of the libraries, Qubit 4.0 (Thermo Fisher Scientific Inc.) and Agilent 2200 Bioanalyzer (Agilent Inc.) were used to detect the concentration and fragment size distribution of the libraries, respectively. The libraries were sequenced on Illumina NovaSeq platforms with paired-end reads of 150 bp.

The raw Illumina sequence data were demultiplexed and converted to FASTQ files, and adapter and low-quality sequences were quantified. Sample reads were then mapped to the hg38 human genome reference using HISAT2. We obtained the fragments per kilobase of transcript per million mapped reads (FPKM) using StringTie and Ballgown. To choose genes with accurate expression values, we removed genes whose FPKM was 0 in more than 30% of the samples before subsequent analyses.

Whole-exome sequencing

Sample preparation and data generation

Qualified genomic DNA from tissues and matched white blood cell samples was prepared for whole-exome sequencing (WES). A total of 300 ng of each DNA sample based on Qubit quantification was fragmented on a Bioruptor Plus sonication system (Diagenode, Liège, Belgium). Sheared DNA was used to perform end repair, A-tailing and adapter ligation with an Agilent SureSelectXT Library Prep Kit (Agilent Technologies, Santa Clara, CA, USA) according to the manufacturer's protocol. Then, 750 ng of prepared DNA in a volume of 3.4 ml was captured using Agilent SureSelect Human All Exon V6 (Agilent Technologies) probes, followed by the amplification of the captured library with indexing primers. Quality control was performed using the Agilent 2100 Bioanalyzer (Agilent Technologies) with a DNA chip. After quantified with a Qubit® 3.0 fluorometer (Invitrogen, Carlsbad, CA, USA), the libraries were sequenced on an Illumina HiSeq platform (Illumina Inc., San Diego, CA, USA). For each library preparation from tissue, 12 samples were loaded in a single lane. For each library preparation from blood, 20 samples were loaded in a single lane.

Genomic data analysis

The exome-sequenced reads were aligned using BWA-mem, and the resulting BAM files were preprocessed with duplicate marking and base quality score recalibration using version 202010.02 of Sentieon Genomics tools [9]. Sequencing quality was assessed using NGSCheckMate [10], FastQ Screen [11], FastQC [12] and Qualimap [13].

Somatic variant calling

VarScan2 v2.4.2 [14] (--min-coverage 3 --min-coverage-normal 3 --min-coverage-tumor 3 --min-var-freq 0.08 --p-value 0.10 --somatic-p-value 0.05 –strand-filter 1), TNseq [9], and TNscope [15] (sentieon driver -t -r --algo TNscope --dbsnp --pon) were used to identify the somatic mutations. Specifically, for the raw VarScan2 results, processSomatic and somaticFilter (--min-coverage 10 --min-reads2 2 2 --min-strands2 1 --min-avg-qual 20 --p-value 0.1) were used to extract high-confidence somatic mutations and to remove clusters of false positives and SNV calls near indels. TNseq detected and filtered out variants by TNhaplotyper2 (--germline_vcf --pon --algo OrientationBias and --algo ContaminationModel) and TNfilter (--contamination --tumor_segments --orientation_priors). Both TNseq and TNscope adopted a panel of

normal (PoN) samples to screen out expected germline variation and artifacts. This PoN panel was based on 699 normal blood samples, from which two VCF files were created for the sites identified as mutations by TNseq and TNscope. In addition, the location of the population germline resource containing the population allele frequencies obtained from gnomAD [16] was used to filter the raw TNseq results.

To obtain the final set of variant calls, we used a two-step approach, first removing any spurious variant calls arising as a consequence of sequencing artifacts and then making use of consensus mutations in at least two out of three callers to identify somatic mutations. Second, additional filtering based on bam-readcount (https://github.com/genome/bam-readcount) was performed to reduce false-positive calls: 1) variant allele frequency (VAF) ≥ 5%; 2) sequencing depth in the region ≥ 8; and 3) sequence reads in support of the variant call ≥ 4. A catalog of cancer driver genes was assembled with: (1) the curated cancer gene list by OncoKB (October 2020) [17]; (2) previously published and functionally validated oncogenic driver genes by Bailey, et al [18]; (3) the compendium of mutational cancer driver genes from the Integrative OncoGenomics [19]; (4) genes recorded as oncogene or tumor suppressor gene (TSG) by the Cancer Gene Census [20].

Copy number alterations

Sample preparation and data generation

The OncoScan CNV Assay Kit (Affymetrix, Santa Clara, CA, USA) was used to perform genome-wide copy number analysis according to the manufacturer's recommendations. Briefly, a total of 80 ng of DNA from each tumor sample was processed. Molecular inversion probes (MIPs) were mixed with the sample DNA and annealed at 58°C overnight. The annealed DNA was divided into two equal parts and incubated with AT or GC gap-fill master mixes for ligation. Then, exonuclease treatment was performed to remove the unincorporated, noncircularized MIPs and the remaining genomic template. The circularized MIPs were linearized with a cleavage enzyme, and the two PCR amplifications were performed successively. The amplified products were digested with HaeIII and Exo enzymes, and the small fragments containing the specific single-nucleotide polymorphism (SNP) genotype were hybridized onto arrays. The arrays were washed and stained using a GeneChip Fluidics Station 450 (Affymetrix, Santa Clara, CA, USA) and scanned using a GeneChip Scanner 3000 7G (Affymetrix, Santa Clara, CA, USA). The fluorescence of clusters was measured to generate a DAT file. Cluster intensity values were automatically calculated using a built-in algorithm from DAT files using GeneChip Command Console software (Affymetrix, Santa Clara, CA, USA), and a CEL file was generated.

Analysis of SNP array data

An analysis of Affymetrix OncoScan CNV SNP probe assays was performed with Chromosome Analysis Suite (ChAS) v4.1 software (Thermo Fisher Scientific). A copy number reference model file was built by using a reference cohort of DNA from 23 randomly selected white blood cell samples from the mentioned patients and positive control samples from the OncoScan CNV Assay Kit. Probe-level output from the ChAS was analyzed using ASCAT (v2.4.3) [21] to obtain segmented copy number calls, estimated tumor ploidy and estimated tumor purity results. The ASCAT segments were subsequently used to produce log2 ratios by dividing by the total copy number (nAraw + nBraw, with zero values set to 0.05). These segments were used as the input of GISTIC2.0 (v2.0.22)[22] to study the recurrence of gene-level CNVs in our sample set. GISTIC2.0 was run with the following parameters changed from the default settings (-ta 0.2 -td 0.2 -genegistic 1 -smallmem 1 -broad 1 -conf 0.95 -rx 0 –brlen 0.7 -cap 3.5 –armpeel 1 -js 100). Moreover, a group of adjacent normal tissues from 23 patients was used to filter recurrent germline/potential false-positive calls. Based on their segment output, the probes that suggested gain or loss in at least five patients were used with the help of Integrative Genomics Viewer to constitute a CNV file for removing recurrent germline/potential false-positive calls in GISTIC2.0.

MS sample processing and data collection for proteomics

Proteome analysis

Proteins were extracted and denatured from 1-2 mg of fresh frozen tissues in 30 µL lysis buffer (6 M urea, 2 M thiourea, 100 mM triethylammonium bicarbonate), followed by proteolytic digestion using Lys-C (Hualishi, Beijing, China) and trypsin (Hualishi, Beijing, China) assisted by pressure-cycling technology (PCT), as described previously [23,24]. TMTpro 16plex label reagents (Thermo Fisher Scientific, San Jose, USA) were used to label the digested peptides. A common pooled peptide sample was used as the reference control sample for each TMT batch. The TMT-labeled samples were cleaned with a C18 column (Waters Sep-Pak® Vac 1 cc C18 Cartridge) and fractionated using a Dionex UltiMate3000 HPLC system (Thermo Fisher Scientific, San Jose, USA) as described previously (Gao et al., 2020). Peptides were separated into 60 fractions, which were then consolidated into 30 fractions. The redissolved peptides were analyzed by liquid chromatography–tandem mass spectrometry (LC–MS/MS) with a nanoflow DIONEX UltiMate 3000 RSLCnano System (Thermo Scientific™, San Jose, USA) coupled with an Orbitrap Exploris 480 mass spectrometer (Thermo Scientific™, San Jose, USA), which was equipped with a FAIMS Pro™ (Thermo Scientific™, San Jose, USA) in data-

dependent acquisition (DDA) mode. The peptide samples were analyzed using an LC gradient of 60 min. The other LC–MS parameters followed a previous publication (Gao et al., 2020).

Database search

The mass spectrometric (MS) data were analyzed by Proteome Discoverer (Version 2.4.1.15, Thermo Fisher) using the human protein database downloaded from UniProt (version 15/07/2020, 20368). Two trypsin missed cleavages were allowed. The minimal peptide length was set to 6 residues. Normalization was performed against the total peptide amount. Carbamidomethylation (+57.021 Da) of cysteine was set as static modification, while oxidation (+15.995 Da) of methionine and acetylation (+42.011 Da) of peptides' N-termini were set as variable modifications. Lysine residues and peptide N-termini were tagged with TMTpro (+304.207 Da). Precursor ion mass tolerance was set to 10 ppm, and fragment mass tolerance was to 0.02 Da. Both unique peptides and razor peptides were used for mapping the best associated master proteins. The master protein abundances were calculated by summation of their associated peptide groups. The false discovery rate (FDR) of peptide was set to 1% (strict) and 5% (relaxed). The other parameters followed the default setup. More details have been described previously [25].

Normalization and quality control of proteomic data

In the primary matrix of proteome data, columns were tested samples and rows were detected proteins. Samples with outlier median intensity ratio [defined as >mean(intensity ratio)+2*sd(intensity ratio) or <mean(intensity ratio)-2*sd(intensity ratio)] were excluded by tumor samples and para-tumor samples respectively. Then, the matrix was first log2-transformed and then normalized by column-median. Batch effects were removed by the removeBatchEffect function in the R package limma [26]. Batch effects before and after batch effect removal were evaluated by principal component analysis (PCA). Proteins that were absent in over 30% of all samples were not included in further quality control analysis. Then samples were filtered by PCA, where sample out of the 90% confidence eclipses in PCA analysis (plotted with PC1 and PC2 using all included proteins) by tumor samples and para-tumor samples respectively. Finally, duplicated samples and genes were merge by using mean value.

MS sample processing and data collection for metabolomics

Polar metabolomics detection

1) Sample quenching and extraction

Twenty-five milligrams of sample was weighed into an EP tube, and 500 μL of extraction solution (methanol:acetonitrile:water = 2:2:1) was added. Then, the samples were homogenized at 35 Hz for 4 min and sonicated for 5 min in an ice-water bath. The homogenization and sonication cycle was repeated 3 times. The samples were incubated for 1 h at -40°C and centrifuged at 12000 rpm for 15 min at 4°C [27]. The QC sample was prepared by mixing equal aliquots of the supernatants from all of the samples.

2) Chromatography separation

LC–MS/MS analyses were performed using an ultrahigh-performance liquid chromatography (UHPLC) system (Vanquish, Thermo Fisher Scientific) with a UPLC BEH Amide column (2.1 mm × 100 mm, 1.7 μm) coupled to a Q Exactive High Field (QE-HFX) mass spectrometer (Orbitrap MS, Thermo). The mobile phase consisted of 25 mmol/L ammonium acetate and 25 mmol/L ammonia hydroxide in water (pH = 9.75) (A) and acetonitrile (B). The autosampler temperature was 4°C, and the injection volume was 2 μL.

3) Mass spectrometry

A QE HFX mass spectrometer was used for its ability to acquire MS and MS/MS spectra in information-dependent acquisition (IDA) mode in the control of the acquisition software (Xcalibur, Thermo). In this mode, the acquisition software continuously evaluates the full-scan MS spectrum. The electrospray ionization (ESI) source conditions were set as follows: sheath gas flow rate of 30 Arb, Aux gas flow rate of 25 Arb, capillary temperature of 350°C, full MS resolution of 60000, MS/MS resolution of 7500, collision energy of 10/30/60 in normalized collision energy (NCE) mode, and spray voltage of 3.6 kV (positive) or -3.2 kV (negative).

4) Data processing, metabolite identification and data analysis

MS raw data files were converted to mzXML format by ProteoWizard software (version 3.0.19282) and processed by the R package XCMS (v3.2) for metabolomics. The data pretreatments include peak identification, peak alignment, peak extraction, retention time correction and peak integration. To make the metabolomics data reproducible, peaks with RSDs larger than 30% in the QC samples were filtered out. The remaining peaks were annotated by comparison to retention time and mass to charge ratio (m/z) indices in the library by using the R package CAMERA [28]. After that, we

obtained a data matrix consisting of the retention time, m/z and peak intensities. The data matrix was further processed by removing the peaks with missing values (intensity = 0) in more than 50% of the samples. For each metabolite, the missing values were replaced with 50% of the lowest observed value of all detected samples [29,30]. The area of each peak was then normalized by isotopically labeled ISs for polar metabolomics [31]. To remove the unwanted intra- and interbatch analytical variations, each metabolite peak in all subject samples was normalized using the locally estimated scatterplot smoothing (LOESS) method based on QC samples [31]. In brief, a LOESS regression model was built based on the intensity drift of each metabolite in the QC samples and used to predict and correct the intensities of the same metabolite in subject samples [31]. In all, 11708 MS features were included for further annotation.

Then the MS/MS spectra were searched in an in-house database for polar metabolite annotation based on accurate mass (m/z, ± 5 ppm), retention time and spectral patterns. The MS/MS spectra matching score was calculated using dot-product algorithm, which take the fragments and intensities into consideration [32]. Metabolites with MS/MS matching score higher than 0.3 were included in our study. In all, 669 MS/MS features were annotated and included in our study.

In summary, only the peaks with MS/MS name and with MS/MS matching score higher than 0.3 were included for further analysis.

Lipidomic detection

1) Sample quenching and extraction

Twenty milligrams of sample was weighed into an EP tube. Two hundred microliters of water and 480 μL of extract solution (MTBE: MeOH= 5: 1) were added sequentially. After 30 s of vortexing, the samples were homogenized at 35 Hz for 4 min and sonicated for 5 min in an ice-water bath. The homogenization and sonication cycle was repeated 3 times. Then, the samples were incubated at -40°C for 1 h and centrifuged at 3000 rpm (RCF=900 (×g), R= 8.6 cm) for 15 min at 4°C. Three hundred microliters of supernatant was transferred to a fresh tube, and the QC sample was prepared by mixing equal aliquots of the supernatants from all of the samples and drying the mixture in a vacuum concentrator at 37°C. Then, the dried samples were reconstituted in 150 μL of 50% methanol in dichloromethane by sonication for 10 min in an ice-water bath. The constitution was then centrifuged at 13000 rpm (RCF=16200 (×g), R= 8.6 cm) for 15 min at 4°C, and 120 μL of supernatant was transferred to a fresh glass vial for LC/MS analysis.

2) Chromatography separation

For lipidomics data collection, LC–MS/MS analyses were performed using a UHPLC system (1290, Agilent Technologies) equipped with a Kinetex C18 column (2.1 * 100 mm, 1.7 μm, Phenomen). Mobile phase A consisted of 40% water, 60% acetonitrile, and 10 mmol/L ammonium formate. Mobile phase B consisted of 10% acetonitrile and 90% isopropanol, to which 50 mL of 10 mmol/L ammonium formate was added for every 1000 mL of mixed solvent. The analysis was carried out with an elution gradient as follows: 0~12.0 min, 40%~100% B; 12.0~13.5 min, 100% B; 13.5~13.7 min, 100%~40% B; and 13.7~18.0 min, 40% B. The column temperature was 55°C. The autosampler temperature was 4°C, and the injection volume was 3 μL (pos) or 3 μL (neg).

3) Mass spectrometry

A QE mass spectrometer was used for its ability to acquire MS and MS/MS spectra in DDA mode in the control of the acquisition software (Xcalibur 4.0.27, Thermo). In this mode, the acquisition software continuously evaluates the full-scan MS spectrum. The ESI source conditions were set as follows: sheath gas flow rate of 30 Arb, Aux gas flow rate of 10 Arb, capillary temperature of 320°C (positive) and 300°C (negative), full MS resolution of 70000, MS/MS resolution of 17500, collision energy of 15/30/45 in NCE mode, and spray voltage of 5 kV (positive) or -4.5 kV (negative).

4) Data processing, metabolite identification and data analysis

MS raw data files were converted to mzXML format by ProteoWizard software (version 3.0.19282) and processed by LipidAnalyzer for lipidomics data. The data pretreatments include peak identification, peak alignment, peak extraction, retention time correction and peak integration. The filtering of reliable lipid peaks and the normalization of data were similar to that in polar metabolomics. Then, the LipidBlast database was applied for lipid annotation. The MS/MS spectra matching score was also calculated as described in the polar metabolomic analysis section. Generally, 1312 MS/MS peaks were identified for lipidomics.

In summary, only the peaks with MS/MS name and with MS/MS matching score higher than 0.3 were included for further analysis.

References

1 Lehmann, B. D. *et al.* Identification of human triple-negative breast cancer subtypes and preclinical models for selection of targeted therapies. *The Journal of clinical investigation* **121**, 2750-2767, doi:10.1172/jci45014 (2011).

2 Burstein, M. D. *et al.* Comprehensive genomic analysis identifies novel subtypes and targets of triple-negative breast cancer. *Clinical cancer research : an official journal of the American Association for Cancer Research* **21**, 1688-1698, doi:10.1158/1078-0432.Ccr-14-0432 (2015).

3 Quist, J. *et al.* A Four-gene Decision Tree Signature Classification of Triple-negative Breast Cancer: Implications for Targeted Therapeutics. *Mol Cancer Ther* **18**, 204-212, doi:10.1158/1535-7163.MCT-18-0243 (2019).

4 Agostinetto, E. *et al.* HER2-Low Breast Cancer: Molecular Characteristics and Prognosis. *Cancers* **13**, 2824 (2021).

5 Comprehensive molecular portraits of human breast tumours. *Nature* **490**, 61-70, doi:10.1038/nature11412 (2012).

6 Schettini, F. *et al.* Clinical, pathological, and PAM50 gene expression features of HER2-low breast cancer. *npj Breast Cancer* **7**, 1-13 (2021).

7 Liu, X. Y. *et al.* Genomic Landscape and Endocrine-Resistant Subgroup in Estrogen Receptor-Positive, Progesterone Receptor-Negative, and HER2-Negative Breast Cancer. *Theranostics* **8**, 6386-6399, doi:10.7150/thno.29164 (2018).

8 Sinn, B. V. *et al.* SET(ER/PR): a robust 18-gene predictor for sensitivity to endocrine therapy for metastatic breast cancer. *NPJ Breast Cancer* **5**, 16, doi:10.1038/s41523-019-0111-0 (2019).

9 Freed, D., Aldana, R., Weber, J. A. & Edwards, J. S. The Sentieon Genomics Tools-A fast and accurate solution to variant calling from next-generation sequence data. *BioRxiv*, 115717 (2017).

10 Lee, S. *et al.* NGSCheckMate: software for validating sample identity in next-generation sequencing studies within and across data types. *Nucleic Acids Res.* **45**, e103-e103 (2017).

11 Wingett, S. W. & Andrews, S. FastQ Screen: A tool for multi-genome mapping and quality control. *F1000Res* **7**, 1338, doi:10.12688/f1000research.15931.2 (2018).

12 Andrews, S. FASTQC: A Quality Control Tool for High Throughput Sequence Data. http://www.bioinformatics.babraham.ac.uk/projects/ (2014).

13 Okonechnikov, K., Conesa, A. & García-Alcalde, F. Qualimap 2: advanced multi-sample quality control for high-throughput sequencing data. *Bioinformatics* **32**, 292-294, doi:10.1093/bioinformatics/btv566 (2016).

14 Koboldt, D. C. *et al.* VarScan 2: somatic mutation and copy number alteration discovery in cancer by exome sequencing. *Genome research* **22**, 568-576 (2012).

15 Freed, D., Pan, R. & Aldana, R. TNscope: accurate detection of somatic mutations with haplotype-based variant candidate detection and machine learning filtering. *bioRxiv*, 250647 (2018).

16 Karczewski, K. & Francioli, L. The genome aggregation database (gnomAD). *MacArthur Lab* (2017).

17 Chakravarty, D. *et al.* OncoKB: A Precision Oncology Knowledge Base. *JCO Precis Oncol* **2017**, doi:10.1200/PO.17.00011 (2017).

18 Bailey, M. H. *et al.* Comprehensive Characterization of Cancer Driver Genes and Mutations. *Cell* **174**, 1034-1035, doi:10.1016/j.cell.2018.07.034 (2018).

19 Martinez-Jimenez, F. *et al.* A compendium of mutational cancer driver genes. *Nat Rev Cancer* **20**, 555-572, doi:10.1038/s41568-020-0290-x (2020).

20 Futreal, P. A. *et al.* A census of human cancer genes. *Nat Rev Cancer* **4**, 177-183, doi:10.1038/nrc1299 (2004).

21 Van Loo, P. *et al.* Allele-specific copy number analysis of tumors. *Proceedings of the National Academy of Sciences of the United States of America* **107**, 16910-16915, doi:10.1073/pnas.1009843107 (2010).

22 Mermel, C. H. *et al.* GISTIC2.0 facilitates sensitive and confident localization of the targets of focal somatic copy-number alteration in human cancers. *Genome biology* **12**, R41, doi:10.1186/gb-2011-12-4-r41 (2011).

23 Guo, T. *et al.* Rapid mass spectrometric conversion of tissue biopsy samples into permanent quantitative digital proteome maps. *Nature medicine* **21**, 407-413, doi:10.1038/nm.3807 (2015).

24 Zhu, Y. *et al.* High-throughput proteomic analysis of FFPE tissue samples facilitates tumor stratification. *Mol Oncol* **13**, 2305-2328, doi:10.1002/1878-0261.12570 (2019).

25 Shen, B. *et al.* Proteomic and Metabolomic Characterization of COVID-19 Patient Sera. *Cell* **182**, 59-72 e15, doi:10.1016/j.cell.2020.05.032 (2020).

26 Ritchie, M. E. *et al.* limma powers differential expression analyses for RNA-sequencing and microarray studies. *Nucleic acids research* **43**, e47, doi:10.1093/nar/gkv007 (2015).

27 Yang, C. *et al.* Effect of vitamin D3 on immunity and antioxidant capacity of pearl oyster Pinctada fucata martensii after transplantation: Insights from LC-MS-based metabolomics analysis. *Fish Shellfish Immunol.* **94**, 271-279, doi:10.1016/j.fsi.2019.09.017 (2019).

28 XueKe, G. *et al.* Lipidomics and RNA-Seq Study of Lipid Regulation in Aphis gossypii parasitized by Lysiphlebia japonica. *Sci. Rep.* **7**, 1364, doi:10.1038/s41598-017-01546-1 (2017).

29 Wei, R. *et al.* Missing Value Imputation Approach for Mass Spectrometry-based Metabolomics Data. *Sci Rep* **8**, 663, doi:10.1038/s41598-017-19120-0 (2018).

30 Tiedt, S. *et al.* Circulating Metabolites Differentiate Acute Ischemic Stroke from Stroke Mimics. *Ann Neurol* **88**, 736-746, doi:10.1002/ana.25859 (2020).

31 Dunn, W. B. *et al.* Procedures for large-scale metabolic profiling of serum and plasma using gas chromatography and liquid chromatography coupled to mass spectrometry. *Nat. Protoc.* **6**, 1060-1083, doi:10.1038/nprot.2011.335 (2011).

32 Stein, S. E. & Scott, D. R. Optimization and testing of mass spectral library search algorithms for compound identification. *J Am Soc Mass Spectrom* **5**, 859-866, doi:10.1016/1044-0305(94)87009-8 (1994).
