# Supplementary material for: Molecular features and clinical implications of the heterogeneity in Chinese patients with HER2-low breast cancer
Source: Nat Commun. 2023 Aug 22;14:5112. doi: 10.1038/s41467-023-40715-x (PMC10444861; doi:10.1038/s41467-023-40715-x)
Supplement: Supplementary file 2 — Reporting Summary [file 41467_2023_40715_MOESM2_ESM.pdf]

nature portfolio

Corresponding author(s): Zhi-Ming Shao, Yi-Zhou Jiang, Ding Ma, Wen-Tao Yang

Last updated by author(s): Jun 10, 2023

Reporting Summary

Nature Portfolio wishes to improve the reproducibility of the work that we publish. This form provides structure for consistency and transparency in reporting. For further information on Nature Portfolio policies, see our Editorial Policies and the Editorial Policy Checklist.

Statistics

For all statistical analyses, confirm that the following items are present in the figure legend, table legend, main text, or Methods section.

n/a	Confirmed	
☐	☒	The exact sample size (*n*) for each experimental group/condition, given as a discrete number and unit of measurement
☐	☒	A statement on whether measurements were taken from distinct samples or whether the same sample was measured repeatedly
☐	☒	The statistical test(s) used AND whether they are one- or two-sided<br>*Only common tests should be described solely by name; describe more complex techniques in the Methods section.*
☐	☒	A description of all covariates tested
☐	☒	A description of any assumptions or corrections, such as tests of normality and adjustment for multiple comparisons
☐	☒	A full description of the statistical parameters including central tendency (e.g. means) or other basic estimates (e.g. regression coefficient) AND variation (e.g. standard deviation) or associated estimates of uncertainty (e.g. confidence intervals)
☐	☒	For null hypothesis testing, the test statistic (e.g. *F*, *t*, *r*) with confidence intervals, effect sizes, degrees of freedom and *P* value noted<br>*Give P values as exact values whenever suitable.*
☒	☐	For Bayesian analysis, information on the choice of priors and Markov chain Monte Carlo settings
☒	☐	For hierarchical and complex designs, identification of the appropriate level for tests and full reporting of outcomes
☒	☐	Estimates of effect sizes (e.g. Cohen's *d*, Pearson's *r*), indicating how they were calculated

Our web collection on statistics for biologists contains articles on many of the points above.

Software and code

Policy information about availability of computer code

Data collection

No custom algorithms or software that were central to this research was used.

RNA sequencing: RNA libraries were sequenced on Illumina NovaSeq platforms with paired-end reads of 150 bp.The raw Illumina sequence data were demultiplexed and converted to FASTQ files, and adapter and low-quality sequences were quantified. Sample reads were then mapped to the hg38 human genome reference using HISAT2 (version 2.1). We obtained the fragments per kilobase of transcript per million mapped reads (FPKM) using StringTie (version 1.3.4) and Ballgown (version 2.14.1).

Whole-exome sequencing: Prepared genomic DNA were sequenced on an Illumina HiSeq platform (Illumina Inc., San Diego, CA, USA). The exome-sequenced reads were aligned using BWA-mem (version 202010.02 of Sentieon Genomics tools), and the resulting BAM files were preprocessed with duplicate marking and base quality score recalibration using Sentieon Genomics tools (version 202010.02). Sequencing quality was assessed using NGSCheckMate (version 1.0.0), FastQ Screen (version 0.12.0), FastQC (version 0.11.8) and Qualimap (version 2.0.0). VarScan2 (version 2.4.2), TNseq (version 202010.02 of Sentieon Genomics tools) and TNscope (version 202010.02 of Sentieon Genomics tools) were used to identify the somatic mutations.

Copy number alterations: The OncoScan CNV Assay Kit (Affymetrix, Santa Clara, CA, USA) was used to perform genome-wide copy number analysis according to the manufacturer’s recommendations. An analysis of Affymetrix OncoScan CNV SNP probe assays was performed with Chromosome Analysis Suite (ChAS) software (Thermo Fisher Scientific, version 4.1). A copy number reference model file was built by using a reference cohort of DNA from 23 randomly selected white blood cell samples from the mentioned patients and positive control samples from the OncoScan CNV Assay Kit. Probe-level output from the ChAS was analyzed using ASCAT (version 2.4.3) to obtain segmented copy number calls, estimated tumor ploidy and estimated tumor purity results. The ASCAT segments were subsequently used to produce log2 ratios by dividing by the total copy number (nAraw + nBraw, with zero values set to 0.05). These segments were used as the input of GISTIC2.0 (version

2.0.22) to study the recurrence of gene-level CNVs in our sample set.

Proteomics: Peptides digested by Lys-C and trypsin were labeled by TMTpro 16plex label reagents (Thermo Fisher Scientific, San Jose, USA). The TMT-labeled samples were cleaned with a C18 column (Waters Sep-Pak® Vac 1 cc C18 Cartridge) and fractionated using a Dionex UltiMate3000 HPLC system (Thermo Fisher Scientific, San Jose, USA). The redissolved peptides were analyzed by liquid chromatography–tandem mass spectrometry (LC–MS/MS) with a nanoflow DIONEX UltiMate 3000 RSLCnano System (Thermo Scientific™, San Jose, USA) coupled with an Orbitrap Exploris 480 mass spectrometer (Thermo Scientific™, San Jose, USA), which was equipped with a FAIMS Pro™ (Thermo Scientific™, San Jose, USA) in data-dependent acquisition (DDA) mode. The mass spectrometric (MS) data were analyzed by Proteome Discoverer (version 2.4.1.15, Thermo Fisher) using the human protein database downloaded from UniProt (version 15/07/2020, 20368).

Polar metabolomics: LC–MS/MS analyzes were performed using an ultrahigh-performance liquid chromatography (UHPLC) system (Vanquish, Thermo Fisher Scientific) with a UPLC BEH Amide column (2.1 mm × 100 mm, 1.7 μm) coupled to a Q Exactive High Field (QE-HFX) mass spectrometer (Orbitrap MS, Thermo). A QE HFX mass spectrometer was used for its ability to acquire MS and MS/MS spectra in information-dependent acquisition (IDA) mode in the control of the acquisition software (Xcalibur, version 4.1, Thermo). MS raw data files were converted to mzXML format by ProteoWizard software (version 3.0.19282) and processed by the R package XCMS (version 3.2) for metabolomics. Then the MS/MS spectra were searched in an in-house database for polar metabolite annotation based on accurate mass (m/z, ± 5 ppm), retention time and spectral patterns.

Lipidomics: LC–MS/MS analyzes were performed using a UHPLC system (1290, Agilent Technologies) equipped with a Kinetex C18 column (2.1 * 100 mm, 1.7 μm, Phenomen). A QE mass spectrometer was used for its ability to acquire MS and MS/MS spectra in DDA mode in the control of the acquisition software (Xcalibur, version 4.0.27, Thermo). MS raw data files were converted to mzXML format by ProteoWizard software (version 3.0.19282) and processed by LipidAnalyzer for lipidomics data. Then, the LipidBlast database (MSDIAL version 4.48) was applied for lipid annotation. The MS/MS spectra matching score was also calculated as described in the polar metabolomic analysis section.

More details were provided in supplementary materials.

Data analysis

No custom algorithms or software that were central to this research was used.

R software 4.0.4 was used for the analyses. Survival analysis was performed using the Kaplan–Meier method and compared with the log-rank test using the R package “survival” (version 3.3-1), and then visualized by the R package “survminer” (version 0.4.9).Differentially expressed genes (DEGs) between HER2-0 and HER2-low breast cancers were determined using the R package “DESeq2” (version 1.30.1) according to official instructions. Enrichment analysis based on DEGs and differentially expressed proteins was performed using the R package “clusterProfiler” (version 4.0.5). The pathway enrichment score for each sample was evaluated using the ‘‘gsva’’ function in the R package ‘‘GSVA’’ (version 1.38.2) according to package instructions. Tumor mutation burden (TMB) was calculated with the R package “maftools” (version 2.6.05). PAM50 subtypes were determined based on the PAM50 classifier. IntClust subtypes were assigned using CNA and RNA data with R package iC10 (version 1.5).Lehmann's subtype and HRD score were calculated with custom code according to corresponding reports described in the manuscript.

The code was available at GitHub (https://github.com/ljdai98/FUSCC_HER2-low_breast_cancer_study).

For manuscripts utilizing custom algorithms or software that are central to the research but not yet described in published literature, software must be made available to editors and reviewers. We strongly encourage code deposition in a community repository (e.g. GitHub). See the Nature Portfolio guidelines for submitting code & software for further information.

Data

Policy information about availability of data

All manuscripts must include a data availability statement. This statement should provide the following information, where applicable:

- Accession codes, unique identifiers, or web links for publicly available datasets
- A description of any restrictions on data availability
- For clinical datasets or third party data, please ensure that the statement adheres to our policy

Database for data collection
The hg38 human genome reference was downloaded from https://genome-idx.s3.amazonaws.com/hisat/grch38_snptran.tar.gz/. Cancer driver genes were derived from OncoKB (https://www.oncokb.org/), Integrative Onco Genomics (https://www.intogen.org/) and Cancer Genomics Consortium (CGC, https://cancer.sanger.ac.uk/cosmic/) dataset. Human protein database was downloaded from Universal Protein (Uniprot, https://www.uniprot.org/). Annotations for lipids were downloaded from LipidBlast database (https://fiehnlab.ucdavis.edu/projects/lipidblast/).

Database for data analysis
The multiomics data and clinical information of the TCGA-BRCA dataset were downloaded from cBioPortal (https://www.cbioportal.org/). Lipid metabolism genes were extracted from the Kyoto Encyclopedia of Genes and Genomes (KEGG) database (https://www.kegg.jp/). Gene lists of PI3K and ERBB2 signaling and REACTOME gene sets were derived from the MSigDB C2 collection (https://www.gsea-msigdb.org/). The multiomics data and clinical information of the TCGA-BRCA dataset used in this study are available in the cBioPortal (https://www.cbioportal.org/)62,63.

Data generated in this study
The WES data, CNA data, RNA sequencing data and metabolome data generated in this study have been deposited in the GSA database (https://ngdc.cncb.ac.cn/gsa/) under accession codes PRJCA017539 [https://ngdc.cncb.ac.cn/bioproject/browse/PRJCA017539]. The TMT-based MS-quantified protein data have been deposited in the iProX (https://www.iprox.cn) under accession codes IPX0006535000 [https://www.iprox.cn/page/project.html?id=IPX0006535000]. Source data are provided with this paper. A minimum dataset this study has been deposited in the Zenodo (https://zenodo.org/) under DOI 10.5281/zenodo.7958124 [https://doi.org/10.5281/zenodo.7958124]. These data were publicly accessible after publication.
Source data are provided with this paper.

Human research participants

Policy information about studies involving human research participants and Sex and Gender in Research.

Reporting on sex and gender	Sex was determined by investigator observation. We do not restrict sex in the recruitment of our study, but due to the disease type we focused, the majority of included patients were female. Specifically, in all patients finally included in our study, only one patient was male. And thus, we do not perform sex-based analyses and our finding mainly apply to female.
Population characteristics	Population characteristics of participants including age, investigator observed sex,menopause status and neoadjuvant therapy status were included. These characteristics of included patients were detailed in Table 1 and supplementary materials.
Recruitment	Chinese patients nationwide from China diagnosed with breast cancer and get treated at the Department of Breast Surgery at FUSCC from January 1, 2013 to December 31, 2014 were retrospectively reviewed. A total of 773 consecutive patients were enrolled according to the following defined criteria: 1) Willing to participate in this study and signed written informed consent; 2) Patients diagnosed with unilateral invasive breast cancer; 3) Central pathologic examination of tumor specimens performed by the Department of Pathology at FUSCC: ER, PR, and HER2 statuses; 4) No evidence of distant metastasis at diagnosis; 5) Sufficient archived tissue available for further investigation.<br><br>HER2-0 status was defined as a HER2 IHC score of 0; HER2-low status was defined as a HER2 IHC score of 1+ or 2+ with ISH-; HER2-positive status was defined as a HER2 IHC score of 2+ with ISH+ or 3+.The HER2 IHC scores were reevaluated for all HER2-negative samples, where some original HER2 IHC slides were unavailable, and thus be excluded. This exclusion did not involve any subject slecetion.<br><br>Bias may exist because of our single-center design, but we avoided self-selection bias thoroughout the whole study. Discussion about the bias were provided in our manuscript in the Discussion section.
Ethics oversight	This study was approved and supervised by the Institutional Review Board of FUSCC (IRB ID: 050432-4-1911D).

Note that full information on the approval of the study protocol must also be provided in the manuscript.

Field-specific reporting

Please select the one below that is the best fit for your research. If you are not sure, read the appropriate sections before making your selection.

[x] Life sciences [ ] Behavioural & social sciences [ ] Ecological, evolutionary & environmental sciences

For a reference copy of the document with all sections, see nature.com/documents/nr-reporting-summary-flat.pdf

Life sciences study design

All studies must disclose on these points even when the disclosure is negative.

Sample size	We recruited all patient satisfying criteria described between 01/01/2013 and 12/31/2014 as described above and thus no sample-size calculation was performed.
Data exclusions	In the rescoring, 66 patients were excluded because of no available historical HER2 IHC slides.
Replication	Most sequencing and molecular profiling was performed once per human sample because of the limited amount of tissue that is available per patient. Technical replicates (samples from 5 patients) were set to verify the reliability of TMT-labeled proteome system by Spearman's correlation analysis, where technical replicates showed good correlation.
Randomization	Randomization was not relevant to this study because we didn't have group allocation in the experiments.
Blinding	Samples were blinded during the sample preparation and sequencing.

Reporting for specific materials, systems and methods

We require information from authors about some types of materials, experimental systems and methods used in many studies. Here, indicate whether each material, system or method listed is relevant to your study. If you are not sure if a list item applies to your research, read the appropriate section before selecting a response.

Materials & experimental systems

n/a	Involved in the study
☒	☐ Antibodies
☒	☐ Eukaryotic cell lines
☒	☐ Palaeontology and archaeology
☒	☐ Animals and other organisms
☒	☐ Clinical data
☒	☐ Dual use research of concern

Methods

n/a	Involved in the study
☒	☐ ChIP-seq
☒	☐ Flow cytometry
☒	☐ MRI-based neuroimaging
